# A comprehensive analysis of methods for assessing polygenic burden on Alzheimer’s disease pathology and risk beyond *APOE*

**DOI:** 10.1093/braincomms/fcz047

**Published:** 2019-12-16

**Authors:** Andre Altmann, Marzia A Scelsi, Maryam Shoai, Eric de Silva, Leon M Aksman, David M Cash, John Hardy, Jonathan M Schott, Alzheimer’s Disease Neuroimaging Initiative

**Affiliations:** 1 Department of Medical Physics and Biomedical Engineering, Centre for Medical Image Computing (CMIC), University College London (UCL), London WC1V 6LJ, UK; 2 Reta Lilla Research Laboratories, Department of Neurodegeneration, Queen Square Institute of Neurology, University College London (UCL), London WC1V 6LJ, UK; 3 UK Dementia Research Institute, University College London (UCL), London WC1V 6LJ, UK; 4 Institute for Health Informatics, University College London (UCL), London WC1V 6LJ, UK; 5 Dementia Research Centre, Queen Square Institute of Neurology, University College London (UCL), London WC1V 6LJ, UK

**Keywords:** Alzheimer’s disease, polygenic risk score, polygenic hazard score, biomarker, imaging genetics

## Abstract

Genome-wide association studies have identified dozens of loci that alter the risk to develop Alzheimer’s disease. However, with the exception of the *APOE*-ε4 allele, most variants bear only little individual effect and have, therefore, limited diagnostic and prognostic value. Polygenic risk scores aim to collate the disease risk distributed across the genome in a single score. Recent works have demonstrated that polygenic risk scores designed for Alzheimer’s disease are predictive of clinical diagnosis, pathology confirmed diagnosis and changes in imaging biomarkers. Methodological innovations in polygenic risk modelling include the polygenic hazard score, which derives effect estimates for individual single nucleotide polymorphisms from survival analysis, and methods that account for linkage disequilibrium between genomic loci. In this work, using data from the Alzheimer’s disease neuroimaging initiative, we compared different approaches to quantify polygenic disease burden for Alzheimer’s disease and their association (beyond the *APOE* locus) with a broad range of Alzheimer’s disease-related traits: cross-sectional CSF biomarker levels, cross-sectional cortical amyloid burden, clinical diagnosis, clinical progression, longitudinal loss of grey matter and longitudinal decline in cognitive function. We found that polygenic scores were associated beyond *APOE* with clinical diagnosis, CSF-tau levels and, to a minor degree, with progressive atrophy. However, for many other tested traits such as clinical disease progression, CSF amyloid, cognitive decline and cortical amyloid load, the additional effects of polygenic burden beyond *APOE* were of minor nature. Overall, polygenic risk scores and the polygenic hazard score performed equally and given the ease with which polygenic risk scores can be derived; they constitute the more practical choice in comparison with polygenic hazard scores. Furthermore, our results demonstrate that incomplete adjustment for the *APOE* locus, i.e. only adjusting for *APOE*-ε4 carrier status, can lead to overestimated effects of polygenic scores due to *APOE*-ε4 homozygous participants. Lastly, on many of the tested traits, the major driving factor remained the *APOE* locus, with the exception of quantitative CSF-tau and p-tau measures.

## Teaser

Altmann et al. compared different approaches to quantify polygenic disease burden for Alzheimer’s disease and their association with a broad range of Alzheimer’s disease-related traits. Beyond the *APOE* locus, polygenic scores were associated with clinical diagnosis, CSF-tau and p-tau and progressive atrophy, but not with amyloid beta pathology.

## Graphical Abstract

**Figure:**
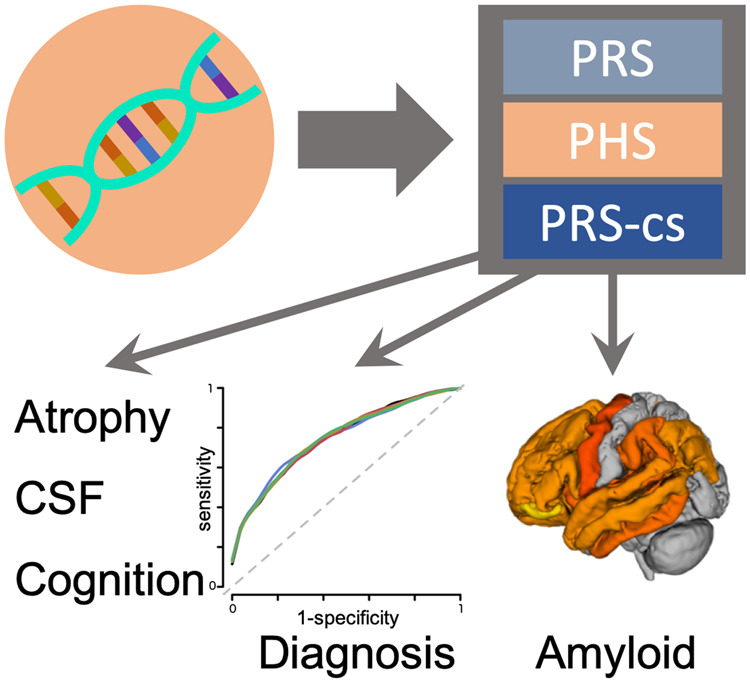

## Introduction

The capability to predict disease risk from a person’s genome has invaluable applications, ranging from stratifying people for interventions, enrolling them in screening programmes to capture the first signs of disease onset and enriching clinical trials. Accurate prediction of both developing disease and approximate timing can be made in familial diseases where a single genetic variant is causative of the disorder, e.g. *APP*, *PSEN1* and *PSEN2* mutations in familial Alzheimer’s disease (Bateman *et al.*, 2012) and the *HTT* trinuclear repeat in Huntington’s disease (MacDonald *et al.*, 1993). The ability to predict disease risk or the age of symptom onset from genetic information is more complex in the sporadic manifestations of various neurodegenerative disorders, despite, in the case of Alzheimer’s disease, it being highly heritable (*h*^2^ range 0.58–0.79; Gatz *et al.*, 2006) and highly polygenic (Kunkle *et al.*, 2019). In the context of late-onset Alzheimer’s disease, with a minor allele frequency of 0.14, the ε4 allele of the *APOE* gene is the strongest common genetic risk factor in people with central European ancestry (odds ratio about 3.5). Consequently, the effect of *APOE*-ε4 on various Alzheimer’s disease biomarkers has been studied extensively over the last decades [reviewed by Belloy *et al.* (2019)] and modifying demographic factors such as ethnicity and sex have been identified (Farrer *et al.*, 1997; Ungar *et al.*, 2013; Altmann *et al.*, 2014).

Despite genetic studies with ever-increasing size (Lambert *et al.*, 2013; Jansen *et al.*, 2019; Kunkle *et al.*, 2019) other variants with equivalent risk to *APOE*-ε4 have failed to materialize. Most variants discovered through genome-wide association studies (GWAS) exhibit only minor effects on Alzheimer’s disease risk (odds ratios between 0.8 and 1.2; Kunkle *et al.*, 2019). One notable exception is the R47H substitution in the *TREM2* gene which confers a similar level of risk to develop Alzheimer’s disease as *APOE*-ε4 (odds ratio about 2.0) but occurs very rarely in the population (global minor allele frequency of 0.0025; Guerreiro *et al.*, 2013). However, if a single variant cannot deliver predictive accuracy equivalent to *APOE*-ε4, then a combination of known risk variants could be of use. This is the rationale behind genome-wide polygenic risk scores (PRSs), which combine the effects of multiple independent risk variants in a person’s genome into a single score capturing an individual’s overall genetic disease risk (International Schizophrenia Consortium *et al.*, 2009). Owing to the ease of access to results from GWAS and the availability of dedicated software (Euesden *et al.*, 2015; Vilhjálmsson *et al.*, 2015), such scores can be built easily and have been adopted rapidly in the genetics field. In recent reports, their capacity to predict disease in common disorders was found to be equivalent to that of (rare) monogenic mutations (Khera *et al.*, 2018). In the context of Alzheimer’s disease PRS have been shown to be associated with clinical diagnosis (Escott-Price *et al.*, 2015), cortical thickness (Sabuncu *et al.*, 2012), memory, hippocampal volume and cognitive decline (Mormino *et al.*, 2016), disease progression (Scelsi *et al.*, 2018) and lastly pathological confirmed cases of Alzheimer’s disease (Escott-Price *et al.*, 2017). Furthermore, Alzheimer’s disease PRSs, which are conceptually based on GWAS of late-onset Alzheimer’s disease, modulate disease risk and age of disease onset in familial late-onset Alzheimer’s disease as well as sporadic early-onset Alzheimer’s disease (Tosto *et al.*, 2017; Cruchaga *et al.*, 2018).

Methods for developing and refining PRS have gained increased attention over the recent years and ever-increasing sample sizes in GWAS result in better-calibrated risk scores for out of sample predictions (Dudbridge, 2013). Beyond these improved effect estimates for single variants, methodological improvements have included the explicit modelling of linkage disequilibrium dependencies between variants (Vilhjálmsson *et al.*, 2015; Ge *et al.*, 2019). A recent conceptual novelty in this domain is the polygenic hazard score (PHS), which seeks to gain predictive power by deriving effect sizes for genetic variants from the time to disease onset in a survival analysis, rather than from wide-spread case–control analyses that are traditionally used (Desikan *et al.*, 2017). A series of recent studies have demonstrated very strong associations between a PHS for Alzheimer’s disease and a range of quantitative measurements related to Alzheimer’s disease pathology including clinical dementia rating (CDR), clinical conversion rates, cognitive tests, cortical *in vivo* amyloid uptake, change in cortical volumes and post-mortem neuropathology (Tan *et al.*, 2017, 2018, 2019). The PHS introduced by Desikan *et al.* (2017) comprises 33 genetic markers (two of which are *APOE*-ε4 and *APOE*-ε2) and is highly correlated with *APOE*-ε4 burden [Supplementary Fig. 1 in Desikan *et al.* (2017)]. Previous works claimed that the PHS is superior to PRS (Tan and Desikan, 2018) and exhibits predictive power beyond *APOE*; however, the published analyses controlled only for *APOE*-ε4 status rather than *APOE*-ε4 allele burden (Desikan *et al.*, 2017; Tan *et al.*, 2017, 2018, 2019).

We hypothesized that by failing to adjust for *APOE* genotype—i.e. adjusting for ε2 and ε4 allele count rather than only for ε4 carrier status—previous reports on the predictive power of PHS over *APOE* were overestimating the impact of PHS. In this work, we investigated whether PRS or PHS are associated with Alzheimer’s disease-related biomarkers and clinical outcomes beyond the *APOE* locus.

## Methods

### Data

Data used in the preparation of this article were obtained from the ADNI database (http://adni.loni.usc.edu). The ADNI was launched in 2003 as a public–private partnership, led by Principal Investigator Michael W. Weiner, MD. The primary goal of the ADNI has been to test whether serial MRI, PET, other biological markers, and clinical and neuropsychological assessment can be combined to measure the progression of mild cognitive impairment (MCI) and early Alzheimer’s disease. For up-to-date information, see www.adni-info.org. ADNI study data were accessed through the R-package ADNIMERGE (accessed: 7 March 2019).

### Preparation of genetic data

At the time of this study, single nucleotide polymorphism (SNP) genotyping data were available for *n* = 1674 subjects across all ADNI phases. Genotyping was conducted using three different platforms: Human610-Quad, HumanOmniExpress and Omni 2.5 M (Illumina; Saykin *et al.*, 2010). Prior to imputation subject-level quality control (QC) steps based on call rate (10% cutoff) and concordance between chip-inferred sex and self-reported sex were performed separately for each genotyping chip. On SNP-level standard QC steps ensuring compatibility with the reference panel used for imputation (strand consistency, allele names, position, Ref/Alt assignments and minor allele frequency discrepancy (MAF; 0.2 cutoff) were conducted. Imputation was carried out using the Sanger Imputation Server (https://imputation.sanger.ac.uk/) with SHAPEIT for phasing (Delaneau *et al.*, 2012), Positional Burrows-Wheeler Transform (Durbin, 2014) for imputation and the entire Haplotype Reference Consortium (release 1.1) reference panel (McCarthy *et al.*, 2016). Data from the three different genotyping platforms were imputed separately. As part of post-imputation QC, multi-allelic variants and SNPs with imputation INFO score <0.3 were removed and genotype calls with posterior probability <0.9 were set to missing (i.e. hard called). Following the initial QC, genotypes from the three platforms were merged. Further information on the imputation and QC process is detailed in Scelsi *et al.* (2018) and https://rpubs.com/maffleur/452627. Using the merged data, we retained SNPs with MAF ≥1% and genotyping rate >0.9.

SNPweights (Chen *et al.*, 2013) was used to infer genetic ancestry from genotyped SNPs using the reference panel comprising Central European, Yoruba Africans and East Asian samples from HapMap 3 (The International HapMap 3 Consortium *et al.*, 2010) and native Americans from Reich *et al.* (2012). Subjects with predicted central European ancestry of 80% or more were retained. Next, using the imputed and merged data genetic relatedness between central European subjects was computed. First, the SNP content was restricted to SNPs with MAF ≥5% and LD-pruning was carried out in PLINK v1.9 (–indep-pairwise 1000 50 0.1). The genetic relatedness matrix was computed using the remaining autosomal SNPs and the dataset was trimmed to remove subjects with relatedness >0.1 (–rel-cutoff 0.1). For the remaining subjects, the first five PCA components were computed in PLINK v1.9 and were used to account for population structure in the data.

### Polygenic risk scores

PRSs were computed using the software PRSice v2.1.9 (Euesden *et al.*, 2015). As base GWAS the stage 1 results of the most recent Alzheimer’s disease GWAS featuring a pure Alzheimer’s disease phenotype was used [Kunkle *et al.*, 2019; this is opposed to a larger GWAS including subjects with parental Alzheimer’s disease diagnosis resulting in diagnosis-by-proxy (Jansen *et al.*, 2019)]. For PRS computation, SNPs with MAF ≥5% were considered and SNPs were selected using LD-clumping (1000 KB, *R*^2^ of 0.1 and *P*-value threshold of 1.0), missing SNPs were simply ignored on subject level (using the setting–missing SET_ZERO) and the *APOE* region was excluded (hg19 coordinates chr19 from 44 400 000 to 46 500 000). Effects for *APOE*-ε2 and *APOE*-ε4 were manually added using effect sizes from (Kunkle *et al.*, 2019): 1.2017 (ε4) and -0.4673 (ε2). For this study, two *P*-value inclusion cut-offs were used: SNPs passing genome-wide suggestive (*P* = 1.0e-05) and *P* = 0.5, which was found to be an optimal choice in an earlier study (Escott-Price *et al.*, 2015). The resulting scores are referred to as PRS1 and PRS2, respectively. In addition to this conventional approach, PRS were generated using posterior effect size estimates generated with PRS-CS (Ge *et al.*, 2019). The effect estimates were generated using the *auto* setting and the PRS are referred to as PRS-cs. PRSs were computed for all ADNI participants with genome-wide genotyping data.

### Polygenic hazard score

The derivation of the PHS was described in detail in Desikan *et al.* (2017). The conceptual difference between PRS and PHS is that PHS uses SNP-level effect size estimates from survival analysis (Cox proportional hazards model) as opposed to odds ratios from case–control analyses. The second difference is that effect sizes in PRS were derived univariately while the hazard ratios used for PHS were derived using a multivariate approach, i.e. effects for each SNP are estimated in the presence of the remaining ones. For this work, we extracted the PHS values provided by the ADNI database (desikanlab table).

### Statistical analyses

A series of association analyses were conducted to measure the effect of PHS on imaging, CSF and cognitive biomarkers in Alzheimer’s disease. First, we aimed to replicate the results on ADNI data presented in Tan *et al.* (2019) by only correcting for *APOE*-ε4 carrier status (i.e. including a variable that takes the values 0 or 1), referred to as ‘PHS (status)’. Next, the association strength of PHS beyond the *APOE* locus was tested by accounting for *APOE*-ε4 and *APOE*-ε2 dosage (i.e. including two variables that take values 0, 1 or 2), referred to as ‘PHS (burden)’. In addition, in absence of a real polygenic score, the effect of *APOE*-ε4 dosage beyond the effect of *APOE*-ε4 carrier status was quantified, referred to as ‘APOE-ε44’. That is, in this setting APOE-ε44 acts as a ‘polygenic’ score that only counts the number of *APOE*-ε4 alleles. Lastly, PHS was compared with standard PRS at two *P*-value cut-offs (1e-05 and 0.5), referred to as ‘PRS1’ and ‘PRS2’, respectively, and PRS-cs. All these methods were adjusted for *APOE* locus. However, previously ADNI contributed 441 samples to the Alzheimer’s Disease Genetics Consortium (ADGC), which in turn contributed to the IGAP study. Base GWAS and target data must be independent from each other to avoid overly optimistic results due to overfitting (Dudbridge, 2013). Thus, *n* = 441 subjects that contributed to the ADGC GWAS were removed for the comparison between PHS and PRS. Furthermore, analyses were restricted to subjects with predicted central European ancestry and self-reported white non-Hispanic ethnicity. Using large *P*-value thresholds (e.g. *P* < 0.05) results in the PRS comprising many SNPs and potentially makes the score more susceptible to geographical differences in genetic structure (Kerminen *et al.*,). To account for this bias in PRS2 and PRS-cs, we additionally tested associations while also adjusting for population structure by including the first five principal components of the genetic relatedness matrix in the corresponding models.

### Disease status

Association between polygenic scores and latest recorded clinical diagnosis were conducted using logistic regression. In particular, four contrasts were explored: CN versus Alzheimer’s disease, CN versus MCI, MCI versus Alzheimer’s disease and CN versus MCI or Alzheimer’s disease. The model was adjusted for sex, years of education and age in addition to *APOE* (either for ‘status’ or for ‘burden’). For subjects with the CN diagnosis the latest recorded age was used; for Alzheimer’s disease subjects the earliest age with recorded Alzheimer’s disease diagnosis was used; for MCI subjects the earliest date of MCI diagnosis was used in the comparison to CN subjects (CN versus MCI and CN versus MCI or Alzheimer’s disease contrasts) and the latest age was used in the comparison with Alzheimer’s disease. In addition to these statistical association tests, we estimated the predictive performance of the logistic regression using 10-fold cross-validation. We used receiver operating characteristics (ROC) curves as well as the area under the ROC curve (AUC) as means to quantify and compare the model performance. Improvement by including polygenic scores over the ‘status’ or the ‘burden’ model was tested using a signed paired Wilcoxon rank-sum test on the 10 AUC estimates obtained from the cross-validation.

### Baseline CSF biomarkers

The ADNI database provides data on Alzheimer’s disease-related CSF biomarkers amyloid β protein fragment 1–42 (Aβ), total tau (tau) and tau phosphorylated at threonine 181 (p-tau). The association between polygenic scores and baseline CSF biomarkers was tested using linear regression models. Prior to the analysis, the biomarker values were log-transformed to render their distribution more normal. Analyses were adjusted for age at baseline visit, sex, years of education and *APOE* (either for ‘status’ or for ‘burden’). Moreover, genetic influence may vary by disease stage, thus linear regression models were estimated for each clinical disease group (CN, MCI and Alzheimer’s disease); effect sizes were combined using the inverse variance method for meta-analyses to obtain an overall effect size and *P*-value. In addition to these statistical association tests, we estimated the predictive performance of the linear regression using 10-fold cross-validation. We used Pearson’s correlation coefficient as means to quantify the model performance. Improvement by including polygenic scores over the ‘status’ or the ‘burden’ model was tested using a signed paired Wilcoxon rank-sum test on the 10 correlation coefficient estimates obtained from the cross-validation.

### Cross-sectional amyloid PET

We followed the analytical setup by Tan *et al.* (2019): associations of the polygenic scores with regional load of amyloid were computed. Amyloid load was derived from PET using the florbetapir tracer (AV45). Florbetapir synthesis and image acquisition details are described in detail elsewhere (http://adni-info.org, Landau *et al.*, 2013). Processed regional AV45 levels were obtained from ‘ucberkeleyav45’ table providing values for cortical regions of the Desikan-Killiany Atlas (Desikan et al., 2006). Regional AV45 levels were divided by AV45 level in the cerebellum region to obtain regional SUVRs. Values for the right and left hemisphere were averaged. For each of the 34 cortical regions, a linear regression was estimated to quantify the effect of polygenic score on regional amyloid load. The models were adjusted for age at imaging, sex, years of education as well as *APOE* (either for ‘status’ or for ‘burden’). In addition to the setup by Tan *et al.* (2019) and as in the case for the CSF biomarkers, an analysis stratified by disease status was carried out. Multiple-testing correction for the tests across the 34 regions was conducted using false discovery rate (FDR).

### Disease progression

Longitudinal data from ADNI were used to quantify the association of polygenic scores with clinical disease progression, i.e. clinical conversion. The effect of polygenic scores on clinical conversion was tested in a Cox proportional hazards model using subjects without a dementia diagnosis at the baseline visit. We defined clinical conversion as a dementia diagnosis in previously not demented participants. A left-truncated (age at entry) right-censored (age at event or last visit) design was chosen (Altmann *et al.*, 2014). The model was adjusted for age at baseline, sex and years of education, *APOE* (either for ‘status’ or for ‘burden’) and stratified for baseline diagnosis (CN or MCI). In addition to these statistical association tests, we estimated the predictive performance of the Cox regression using 10-fold cross-validation. We used the concordance index, which is conceptually related to AUC, as means to quantify the model performance. Improvement by including polygenic scores over the ‘status’ or the ‘burden’ model was tested using a signed paired Wilcoxon rank-sum test on the 10 concordance indices obtained from the cross-validation.

### Cognitive decline

We followed the analytical setup by Tan *et al.* (2019): The effect of polygenic scores on longitudinal measures of cognitive performance was assessed using linear mixed-effects models in non-demented people. In particular, the longitudinal development of clinical dementia rating sum of boxes (CDR-SB) obtained from ADNIMERGE and composite scores for memory (MEM) and executive function (EF) derived using item response theory methods (Crane *et al.*, 2012) and obtained through ADNIMERGE (table uwnpsychsum). The first model was exactly specified as in Tan *et al.* (2019), where the target variable was the change from the baseline cognitive measurement (Δc) and the fixed effects were interactions between time since baseline (*t*) and sex, years of education, age, volume of the entorhinal cortex, amyloid burden in the frontal lobe, *APOE* (either for ‘status’ or for ‘burden’) and PHS and a random intercept for each subject. The variable of interest was the interaction of time and PHS. This model is referred to as ‘intercept only’. In addition, a linear mixed-effects model that included random effects for subject-level decline (random slopes) was used. Model likelihoods were compared with determine which model provides the better fit for the data. Random slope models have been previously used to explore longitudinal changes in ADNI (Holland *et al.*, 2013). Moreover, we added fixed effects that did not interact with time as well as time itself as a fixed effect. These full models were compared with models lacking the polygenic score-by-time interaction using a likelihood ratio test to assess the effect of polygenic scores on longitudinal cognitive decline.

### Longitudinal decline in grey matter

The longitudinal change in grey matter loss was quantified using the boundary shift integral (BSI; Leung *et al.*, 2010). We accessed precomputed BSI values through ADNIMERGE (foxlabbsi table). BSI measures from volumetric T1-weighted MRIs were used that did not use the accelerated acquisition. Moreover, if for the same baseline/repeat pair a set of 1.5T as well as a set of 3.0T scans were available, the 3.0T scan was used. The association between BSI and polygenic scores was tested using a linear mixed-effects model as described above (with random intercepts and slopes). In particular, the BSI represents directly the Δc value, the model was adjusted for age, sex, years of education, *APOE* (either for ‘status’ or for ‘burden’) as well as their interactions with time since baseline. In addition to the whole brain BSI (BBSI), which was derived using the KN-BSI method (Leung *et al.*, 2010) (KMNDBCB in the foxlabbsi table), BSI for the ventricles (VBSI), left (HLBSI) and right (HRBSI) hippocampi were tested as well.

### Data availability

Data used in the preparation of this article were obtained from the ADNI database (http://adni.loni.usc.edu) and are freely available after registration. Derived polygenic scores (PRS1, PRS2 and PRS-cs) and R-scripts to analyse the data and to produce the results presented here are available at https://github.com/andrealtmann/polygenic_AD/

## Results

Whole genotyping data were available for *n* = 1674 Alzheimer’s Disease Neuroimaging Initiative (ADNI) participants spread over three different genotyping platforms. QC and imputation were carried out per platform and were merged into a single file. PRSs were computed for all ADNI participants with genotyping data using the stage 1 results from a recent Alzheimer’s disease GWAS (Kunkle *et al.*, 2019) at *P*-value cut-offs of 1e-05 (PRS1) and 0.5 (PRS2), resulting in PRS based on 55 and 101 450 SNPs, respectively. We also computed a PRS using the novel Bayesian regression and shrinkage priors (PRS-cs) method (Ge *et al.*, 2019). PHS were obtained from the corresponding table in ADNIMERGE provided by Desikan *et al.* (2017). A total of 157 subjects were removed on the basis of non-European ancestry as inferred by SNPweights (Chen *et al.*, 2013), and, *n* = 15 subjects were removed due to relatedness. Next, only those participants who self-reported as white non-Hispanic/Latino ethnicity were retained (*n* = 49 removed). Finally, *n* = 48 subjects lacking a PHS were excluded from the analyses, leaving a total of 1405 eligible ADNI participants for this study. The baseline demographics for the entire cohort are listed in Table 1.

**Table 1:** Baseline demographics of the full cohort

	CN	MCI	Alzheimer’s disease	Total	*P*-value (*F*-value)
N	417	712	275	1404	
Female (%)	205 (49)	279 (39)	118 (43)	603 (43)	0.004 (5.37)
Age (SD)	74.5 (5.6)	73.1 (7.5)	75.1 (7.7)	73.9 (7.1)	4.28e-05 (10.13)
*APOE*-ε4 (0/1/2)	297/110/10	347/286/79	90/134/51	735/530/140	<2e-16 (64.67)
MMSE (IQR)	29 (29–30)	28 (26–29)	23 (22–25)	28 (26–29)	<2e-16 (1061)
Education	16.48 (2.64)	16.02 (2.84)	15.27 (2.92)	16.01 (2.83)	2.37e-07 (15.42)
Amyloid PET (−/+/NA)	146/76/195	171/216/325	14/107/154	331/399/674	<2e-16 (53.24)

Comprising *n* = 1404 individuals after QC of the genetic data. Age given as mean, MMSE given as median, years of education as mean, amyloid PET as negative (−) or positive (+) and unavailable (NA). CN = cognitively normal; MCI = mild cognitive impairment.

The 441 ADNI subjects who contributed to the Alzheimer’s Disease Genetics Consortium (ADGC), which fed into stage 1 of the recent GWAS (Kunkle *et al.*, 2019) were excluded from analyses involving the PRS (at either cut-off) and PRS-cs; affecting *n* = 410 participants in the dataset after the detailed QC. Baseline demographics for the reduced cohort are listed in Supplementary Table 1. We could confirm a very high degree of correlation between PHS and PRS1 (*r*^2^ = 0.75, Supplementary Fig. 1) as previously reported by Leonenko *et al.* (2019*b*), whereas there was no correlation between either PRS2 and either PHS or PRS1 (*r*^2^ ≤ 0.018). PRS-cs was highly correlated with PHS and PRS1 (*r*^2^ ≥ 0.57) and moderately correlated with PRS2 (*r*^2^ = 0.17). Moreover, PHS, PRS1 and PRS-cs exhibited a strong grouping effect by *APOE*-ε4 burden, which was not observed for PRS2 (Supplementary Fig. 1). This grouping effect is reflected in the correlation between *APOE*-ε4 burden and PHS (*r*^2^ = 0.84), PRS1 (*r*^2^ = 0.72) and PRS-cs (*r*^2^ = 0.58). PRS2 was not correlated with *APOE*-ε4 burden (*r*^2^ = 0.017), but instead showed correlation with the first four principal components of population structure (*r*^2^ > 0.05); max *r*^2^ for any of the other polygenic scores with any principal component was 0.0073.

### Polygenic scores are associated with clinical diagnosis beyond *APOE*

The association between polygenic scores and most recent diagnosis was tested for four contrasts using logistic regression. After adjusting for age, sex, years of education and *APOE*-ε4 carrier status, PHS (status) showed a strong association in all three contrasts involving CN subjects (*P* ≤ 4.32e-06), while there was only a nominally significant effect between MCI and Alzheimer’s disease (*P* = 0.044; Table 2). *APOE*-ε4 burden (i.e. number of ε4 alleles), in addition to *APOE*-ε4 status, showed the same pattern, although at a reduced magnitude: *P* ≤ 7.74e-03 for contrasts involving CN subjects and *P* = 0.306 for the association between MCI and Alzheimer’s disease, confirming that *APOE*-ε4 burden was predictive of diagnosis beyond *APOE*-ε4 status. Owing to this pronounced effect of *APOE*-ε4 homozygous carriers, the association of PHS with diagnosis was tested after comprehensive adjustment for the *APOE* locus, i.e. accounting for *APOE*-ε4 burden as well as *APOE*-ε2 burden. The association of PHS (burden) was markedly reduced but remained significant for contrasts involving CN subjects *P* ≤ 1.54e-03; and at trend level for the contrast between MCI and Alzheimer’s disease (*P* = 0.092; Table 2). Inclusion of PHS in the model using *APOE* status improved predictive performance in all four settings (AUC improvement ranging from 0.015 to 0.025; Supplementary Table 2 and Fig. 2). Adding PHS to the model that uses *APOE* burden led only to statistically significant improvement for the CN versus Alzheimer’s disease classification (AUC increased from 0.765 to 0.777; *P* < 0.006; Supplementary Table 2 and Fig. 2).

**Table 2:** Association of polygenic scores with most recent diagnosis

	Score	CN|AD	CN|MCI	MCI|AD	CN|MCI+AD
Entire cohort (*n* = 1404)	PHS (status)	9.46E-10	4.32E-06	0.044	1.48E-09
	PHS (burden)	8.45E-09	1.54E-03	0.092	2.21E-05
	APOE-ε44	6.50E-04	7.74E-03	0.30	5.99E-04
Reduced cohort (*n* = 994)	PHS (burden)	1.28E-03	6.75E-05	0.32	8.49E-05
	PRS1	0.028	7.78E-03	0.68	5.99E-03
	PRS2	0.031	2.91E-04	0.14	4.98E-04
	PRS2*	4.12E-07	8.26E-03	0.010	3.44E-05
	PRS-cs	7.02E-03	2.80E-05	0.30	3.06E-05
	PRS-cs*	2.69E-04	7.37E-04	0.43	2.80E-05

Results show the uncorrected *P*-values obtained from logistic regression while adjusting for age, sex, education and *APOE* (either for ‘status’ or for ‘burden’). Scores with * are additionally adjusted for five principal components reflecting population structure. Reduced cohort does not include subjects who contributed to ADGC. CN = cognitively normal; MCI = mild cognitive impairment; AD = Alzheimer’s disease.

After removal of ADGC participants, PHS (burden) was compared with PRS1, PRS2 and PRS-cs on the same dataset including comprehensive modelling of the *APOE* locus for all scores. PHS on the reduced dataset showed comparable results to the full dataset, i.e. significant *P* ≤ 1.28e-03 effects for contrasts involving CN subjects. PRS1 and PRS2 both replicated the pattern of associations seen with PHS: associations on contrasts including CN (*P* ≤ 0.031) but not detectable between MCI and Alzheimer’s disease (*P* ≥ 0.14). PRS-cs showed *P*-values of the same magnitude as PHS (burden). After adjusting the model for PRS2 in addition for population structure the association with diagnosis was more pronounced (*P* = 4.12e-07 with population structure compared with *P* = 0.031 without) for the other contrasts no such pronounced effect was observed. The PRS-cs model with additional adjustment for population structure showed slightly improved *P*-values over the model without adjustment. However, owing due to only a marginal change in results, the adjustment for population structure in the PRS-cs model was not investigated further.

Predictive performance over the model using *APOE* burden was improved by adding PHS when classifying CN versus MCI (AUC increase of 0.018) or by including PHS or PRS-cs when classifying CN versus MCI or Alzheimer’s disease (AUC increase of 0.011 or 0.02; Supplementary Table 2 and Fig. 2).

### Polygenic scores are associated with CSF tau but not CSF Aβ beyond *APOE*

The association of polygenic scores with log-transformed baseline CSF biomarkers (Aβ, tau and p-tau) was tested using linear models stratified by disease group. When adjusting only for *APOE* ε4 status (i.e. ε4+/−) PHS (status) showed a strong association with baseline CSF Aβ levels (Table 3; *P* = 9.99e-07). However, the effect was driven by homozygous *APOE*-ε4 carriers (APOE-ε44, *P* = 2.54e-10). After comprehensive adjustment for the *APOE* locus, the association between PHS (burden) and Aβ disappeared (*P* = 0.87). This held true also on the reduced dataset for PHS (burden) as well as PRS1, PRS2 and PRS-cs (Table 3; *P* > 0.31). In contrast, the effect of polygenic scores (PHS and PRS) on either tau and p-tau was not driven by *APOE*-ε4 homozygotes (*P* ≥ 0.067). PHS (status) showed significant associations with both CSF tau (*P* = 1.06e-03) and p-tau levels (*P* = 9.25e-04), which remained significant after comprehensive adjustment for the *APOE* locus in PHS (burden) (*P* < 3.15e-03). This held true also for the PHS (burden) and PRS1 on the reduced dataset (Table 3). These associations were corroborated by the performance of the predictive models where adding PHS and PRS1 to the *APOE* burden baseline model improved the model for tau and p-tau but not Aβ (Supplementary Table 3).

**Table 3:** Associations between polygenic scores and CSF biomarkers Aβ, total tau and phosphorylated tau

Sample size (*N*)	Score	Aβ	Tau	p-tau
1008	PHS (status)	9.99E-07	1.06E-03	9.25E-04
	PHS (burden)	0.87	2.33E-03	3.15E-03
	APOE-ε44	2.54E-10	0.067	0.073
807	PHS (burden)	0.37	1.04E-03	6.85E-04
	PRS1	0.31	1.33E-04	5.96E-05
	PRS2	0.49	0.053	0.087
	PRS2*	0.42	0.95	0.95
	PRS-cs	0.82	0.15	0.10

Biomarker values were log-transformed. The table depicts uncorrected *P*-values from linear models adjusted for age, sex, education and *APOE* (either for ‘status’ or for ‘burden’). Results in the top part are for the entire cohort (*n* = 1008) and in the bottom part after excluding ADGC subjects (*n* = 807). The model indicated with * was additionally adjusted for genetic population structure.

### Polygenic scores show only moderate association with cortical amyloid beyond *APOE*

**Figure 1:**
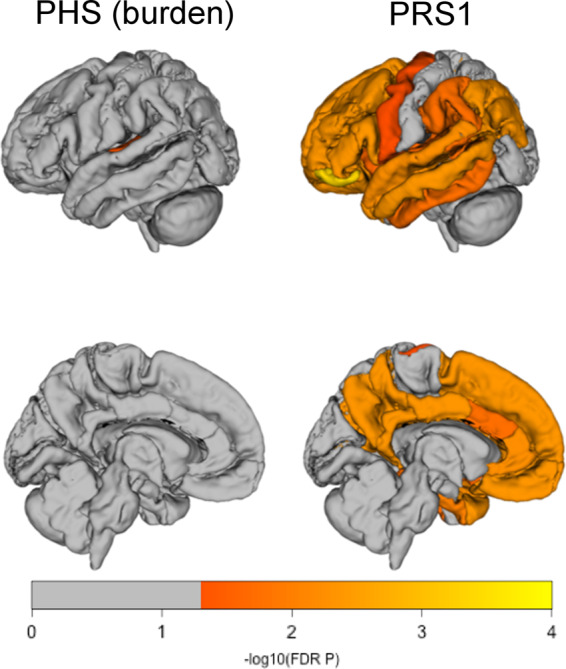
**Regional associations between polygenic risk scores and regional uptake of amyloid.** The colour code represents the -log10 FDR-corrected *P*-values obtained from linear models adjusted for age, sex, education, *APOE*-ε4 count, *APOE*-ε2 count and stratified by disease group at the time of imaging. There were no associations with *P*_FDR_ < 0.05 for PRS2 and PRS-cs.

In addition to CSF Aβ_1__–__42_, cortical amyloid load was quantified using Florbetapir (^18^F) PET (AV45) and tested for association with polygenic scores for Alzheimer’s disease. There were *n* = 917 subjects with AV45 scans (730 obtained at baseline visit). When adjusting for *APOE*-ε4 status there were significant associations between PHS (status) and regional amyloid in all 34 cortical ROIs (*P*_FDR_ range from 1.41e-03 to 6.22e-07; Supplementary Fig. 3 and Table 4) confirming earlier results by Tan *et al.* (2019). Similar to CSF amyloid, cortical amyloid was significantly associated with *APOE*-ε4 burden over *APOE*-ε4 status (28 of 34 regions with *P*_FDR_ from 0.035 to 6.65e-04; Supplementary Fig. 3 and Table 4). Consequently, comprehensive adjustment for the *APOE* locus reduced the association strength of PHS (burden) with regional amyloid, but all of the 34 ROIs remained significantly associated (*P*_FDR_ < 0.0478; Supplementary Fig. 3 and Table 4). Additional stratification by disease status at amyloid imaging, left only one significant ROI with an *P*_FDR_ = 0.0444: transverse temporal gyrus; another 26 ROIs were associated at trend level (*P*_FDR_<0.1; Supplementary Fig. 3 and Table 4). This pattern remained unchanged on the reduced dataset without the ADGC participants (Fig. 1; Supplementary Table 5) comprising *n* = 822 participants with AV45 scans (730 obtained at baseline visit). On the same dataset, PRS1 showed consistent association with regional amyloid despite comprehensive adjustment for *APOE* burden and stratification by disease group (26 of 34 ROIs; *P*_FDR_ from 0.0446 to 4.00e-04; Fig. 1; Supplementary Table 5). Neither, PRS-cs nor PRS2 (with or without correction for population structure) showed any association with regional amyloid load (Supplementary Table 5).

**Table 4:** Effect of polygenic scores on clinical conversion obtained from Cox proportional hazards models

Sample size (N)	Score	Beta	SE	*P*-value
1092	PHS (status)	0.290	0.09	0.002
	PHS (burden)	0.372	0.15	0.013
	APOE-ε44	0.295	0.17	0.088
813	PHS (burden)	−0.005	0.20	0.99
	PRS1	−0.029	0.15	0.84
	PRS2	−0.016	0.08	0.84
	PRS2*	0.290	0.11	0.012
	PRS-cs	−0.181	0.12	0.11

Clinical conversion was defined as a dementia diagnosis in previously not demented participants. Rows correspond to different polygenic scores and estimates for the full dataset (top) and after removal of ADGC subjects (bottom). Beta = log hazards ratio; SE = standard error. *was additionally adjusted for genetic population structure.

### Clinical conversion

The longitudinal design of the ADNI study was leveraged to test the association of polygenic scores with clinical progression to Alzheimer’s disease in non-demented participants using Cox proportional hazards models. There were 1092 non-demented subjects (406 CN, 686 MCI) of whom 318 converted to Alzheimer’s disease (29 CN, 289 MCI). When adjusting for *APOE*-ε4 status in the Cox regression, PHS (status) showed a significant effect on clinical progression (*P* = 0.002), with higher polygenic hazard increasing the risk of conversion, while *APOE*-ε4 burden showed a trend over *APOE*-ε4 status (*P* = 0.09). Comprehensive adjustment for the *APOE* locus led to a decline in association of PHS (burden) with clinical conversion but remained significant (*P* = 0.013). On the reduced dataset, there were 813 subjects (258 CN, 555 MCI) of whom 179 converted to Alzheimer’s disease (11 CN, 168 MCI). None of the scores showed a significant association with clinical conversion (*P* ≥ 0.11, Table 4). However, PRS2 adjusted for population structure showed an association with clinical conversion (*P* = 0.012; Table 4). None of the polygenic scores improved the predictive performance of the baseline models in the Cox regression (Supplementary Table 6).

### Cognitive decline

The effect of polygenic burden on cognitive decline was assessed on 4465 observations obtained from 601 non-demented subjects (220 CN, 381 MCI) using linear mixed effect models with an initial setup as described in Tan *et al.* (2019) followed by a more refined model (see Methods section). Initially, in a model adjusted for *APOE*-ε4 status and using only random intercepts PHS (status) showed a very strong association with decline in CDR-SB (*P* = 7.8e-09), memory (*P* = 8.15e-03) and EF (*P* = 1.32e-03) replicating earlier results (Tan *et al.*, 2019). Using the same model with only random intercepts, *APOE*-ε4 burden exhibited effects of similar strength (Table 5). Consequently, after comprehensive adjustment for *APOE*, PHS (burden) only showed moderate association with CDR-SB (*P* = 3.95e-03) and none for memory or EF (*P* > 0.10). Using a linear mixed-effects model with random slopes and random intercepts (while adjusting only for *APOE*-ε4 status), substantially reduced the association between PHS (status) and CDR-SB (*P* = 5.92e-03), memory (*P* = 0.012) and marginally for EF (*P* = 3.7e-03) compared with random intercepts model (Table 5). Models using random slopes and random intercepts showed significantly higher likelihood (*P* < 2.2e-16) than models using only random intercepts (Supplementary Table 7). Combining comprehensive adjustment for the *APOE* locus and the linear mixed effects model with random slopes and random intercept left no significant association (*P* ≥ 0.08) between the PHS (burden) and decline in cognitive scores. On the same dataset (i.e. there were no ADGC participants), PRS1 showed a moderate significant association with decline in CDR-SB (*P* = 3.57e-03) but not with decline in memory or EF (*P* ≥ 0.42). There was neither a significant association for PRS2 (*P* ≥ 0.21) nor for PRS-cs (*P* ≥ 0.34). After additional adjustment for population structure, PRS2 showed a trending association with decline in CDR-SB (*P* = 0.059) but neither with memory nor with EF decline (*P* ≥ 0.40).

**Table 5:** Associations between polygenic scores and cognitive decline

Model	Score	CDR-SB	MEM	EF
Random intercept	PHS (status)	7.80E-09	8.15E-03	1.32E-03
	PHS (burden)	3.95E-03	0.20	0.10
	APOE-ε44	2.09E-08	2.02E-03	9.19E-05
Random intercept + slope	PHS (status)	5.92E-03	0.012	3.70E-03
	PHS (burden)	0.080	0.47	0.55
	PRS1	3.57E-03	0.42	0.65
	PRS2	0.45	0.21	0.71
	PRS2*	0.059	0.40	0.43
	PRS-cs	0.34	0.66	0.65

*P*-values for the score-by-time interaction on change in CDR sum of boxes) and composite scores for memory and executive function. Rows correspond to different polygenic scores. Linear mixed-effects models were estimated with either random intercept or random intercept and random slope (for time). CDR-SB = clinical dementia rating sum of boxes; MEM = memory; EF = executive function. *was additionally adjusted for genetic population structure.

### Grey matter decline

The BSI (Leung *et al.*, 2010) was used to quantify longitudinal decline in grey matter for the whole brain, the ventricles as well as left and right hippocampus. On the entire dataset, there were *n* = 1250 subjects with 3909 scans for whole brain and ventricular BSI. A reduced number of subjects (*n* = 618) and scans (*n* = 1714) was available for hippocampal BSI. Using a linear mixed-effects model with random intercepts and random slopes, there was a strong association between PHS (status) and whole brain and ventricular BSI (*P* ≤ 2.38e-05) and trending for hippocampal BSI (*P* ≤ 0.091). Some of these effects were driven by *APOE*-ε4 homozygotes (Table 6) and consequently, the association between PHS (burden) and BSI measures was reduced (*P* ≤ 4.12e-03 for whole brain and ventricular; *P* ≥ 0.3 for hippocampi). On the dataset lacking the ADGC participants, there was no significant association between PHS (burden) or PRS2 and any BSI measures (*P* ≥ 0.19). PRS1 was associated with whole brain and ventricular BSI (*P* ≤ 1.7e-03) and marginally for right hippocampus and trending for left hippocampus (Table 6). Similarly, PRS-cs showed at least trend-level association (*P* ≤ 0.061) for any BSI.

**Table 6:** Associations between polygenic scores and atrophy

Subjects (scans)	Score	BBSI	VBSI	HRBSI*	HLBSI*
*n* = 1250 (*n* = 3909)	PHS (status)	1.54e-5	2.38e-5	9.14e-2	5.45e-2
	PHS (burden)	2.56e-3	4.12e-3	0.82	0.30
	APOE-ε44	4.93e-3	1.50e-2	1.25e-2	2.96e-2
*n* = 878 (*n* = 2754)	PHS (burden)	0.19	0.42	0.82	0.30
	PRS1	6.7e-4	1.7e-3	0.02	0.065
	PRS2	0.65	0.91	0.88	0.29
	PRS2**	0.02	7.7e-3	0.10	0.15
	PRS-cs	0.0041	0.031	0.061	0.004

*P*-values for a time-by-polygenic score interaction effect on longitudinal changes in grey matter quantified by the BSI. Columns correspond to different target regions and/or measures of BSI. BBSI = whole brain BSI; VBSI = Ventricular BSI; HRBSI = Right side hippocampal BSI; HLBSI = Left side hippocampal BSI. * indicates a reduced dataset with *n* = 618 subjects and *n* = 1714 scans. ** was additionally adjusted for genetic population structure.

## Discussion

In a series of statistical tests, we investigated whether polygenic scores are associated with a range of Alzheimer’s disease-related traits beyond the *APOE* locus. We found that polygenic scores were associated beyond *APOE* with clinical diagnosis, CSF-tau levels and, to a minor degree, with progressive atrophy. However, for many other tested traits such as clinical disease progression, CSF amyloid, cognitive decline and cortical amyloid load, the additional effects of polygenic scores beyond *APOE* were of minor nature. Our results demonstrate that incomplete adjustment for the *APOE* locus can lead to severely overestimated effects of polygenic scores due to *APOE-*ε4 homozygous participants. For instance, while our analysis confirmed the PHS’ association with a range of Alzheimer’s disease-related measures beyond *APOE*-ε4 carrier status first reported by Tan *et al.* (2019), these effects mostly disappeared when using a comprehensive correction for the *APOE* locus comprising the number of *APOE*-ε2 and *APOE*-ε4 alleles, mainly owing to the high correlation between PHS and *APOE*-ε4 burden. Conversely, on the same cohort of subjects, we tested a simple ‘polygenic’ score comprising only the *APOE*-ε4 allele count and could demonstrate its statistical effects beyond *APOE*-ε4 carrier status. Thus, taken together these results suggest that the previously observed associations of PHS with Alzheimer’s disease-related measures (Tan *et al.*, 2017, 2018, 2019) were partially driven by *APOE*-ε4 homozygous participants. Our analysis also uncovered a second source of statistical modelling that led to overestimated effects of PHS on longitudinal changes in cognition and atrophy. The linear mixed-effects models used in PHS analyses (Desikan *et al.*, 2017; Tan *et al.*, 2017, 2018, 2019) only allowed for subject-specific intercepts, i.e. requiring that the rate of decline is shared between all subjects regardless of disease group, baseline characteristics and genetics, and only modulated by the few variables included as fixed effects. More flexibility is provided by allowing individual rates of decline that are subject-specific (random slopes) in addition to random intercepts. Such random slope linear mixed-effects models have been used recently to analyse longitudinal changes in ADNI data (Holland *et al.*, 2013; Mormino *et al.*, 2016) and are likely to more accurately model ‘real-life’ scenarios where individuals decline at very heterogenous rates. Indeed, the addition of random slopes resulted in a significantly better model fit (*P* < 2.2e-16) on our data than models using only random intercepts (Supplementary Table 7). Here, using random slopes in addition to random intercepts led to dramatically reduced associations between PHS and longitudinal changes in cognitive decline. In combination with a corrected adjustment for *APOE*-ε4 burden, this led to a complete lack of association between the PHS-by-time interaction and CDR-SB (*P* = 0.08), which showed initially a very strong association (*P* = 7.80E-09) with the same setup as in Tan *et al.* (2019) (who reported *P* = 2.44e-10).

Overall, we found that PHS and PRS1, which was based on SNPs exceeding the genome-wide suggestive threshold (*P* = 1e-05), were highly correlated (*r*^2^ = 0.75; Supplementary Fig. 1). This confirms earlier results comparing PHS and PRS, which reported that despite the different nature of the statistical analysis, the estimated effects per SNP were very similar (Leonenko *et al.*, 2019*b*). Consequently, the association profile of the two scores was quite similar across the tested Alzheimer’s disease-related measures. In addition to PRS1, we tested two other PRS scores: PRS2, which was based on SNPs exceeding the *P* = 0.5 threshold, and PRS-cs using a novel method that adjust GWAS-derived effect size estimates for local LD structure. PRS-cs was based on all available SNP data, making it in theory more similar to PRS2, but the correction effect, which emphasized variants with strong effects, rendered the final score to be more correlated to PRS1 rather than to PRS2.

PRS2 showed poorer association with traits compared with PRS1. However, PRS2 was quite sensitive to the correction of population structure (as reflected by substantial correlations between PRS2 and principal components reflecting population structure). The effect was most pronounced when testing for an association with clinical diagnosis where the *P*-value improved from *P* = 0.031 (without correction) to *P* = 4.12e-07 (with correction) in the CN versus Alzheimer’s disease contrast (Table 2). A recent study in the Finnish population demonstrated that polygenic scores are sensitive to geographic patterns even in relatively homogenous populations (Kerminen *et al.*, 2019). Furthermore, the observed bias increased with the inclusion of more variants in the polygenic scores. Thus, while PRS1 (and PHS) may be relatively robust to population bias, PRS2 is more sensitive but requires additional correction for population structure. Overall PRS-cs was less biased by this effect, probably owing to the emphasis on high effect size variants, which is expressed by the high correlation with PHS and PRS1.

After comprehensive adjustment for the *APOE* locus and stratification for disease group, there was no association between polygenic scores and amyloid (measured in CSF or through amyloid PET). In initial reports, PHS showed a strong association with cortical amyloid (Tan *et al.*, 2019, with strongest association seen in Rostral Middle Frontal gyrus; *P*_FDR_ = 5.82E-08; replicated in this study with *P*_FDR_=6.21e-07). Implementing adjustments for *APOE* locus and disease status, only one ROI remained significant (Transverse Temporal gyrus, *P*_FDR_ = 0.038). Likewise, CSF Aβ did not show any association with polygenic scores beyond *APOE* (*P* > 0.34). Only CSF tau and p-tau were consistently associated with polygenic risk beyond *APOE* and clinical diagnosis, with the strongest effects for PHS and PRS1. We found that the genes contributing to PRS1 were significantly enriched (*P* = 4.1e-04) among the protein-interaction partners of tau (Supplementary results). The association with tau rather than amyloid pathology confirms recently published results by Leonenko *et al.* (2019*a*) showing that amyloid pathology is mainly driven by *APOE* genotype and that polygenic risk contributes to risk further conversion to Alzheimer’s disease. Moreover, recent works suggest that brain amyloid deposition is driven by the *APOE* locus (Yan *et al.*, 2018) and that a genome-wide significant variant in the Alzheimer’s disease risk gene *BIN1* (rs744373) is associated with tau pathology instead of amyloid pathology (Franzmeier *et al.*, 2019).

For some analyses, we conducted predictive modelling to assess the effect of polygenic scores in addition to the statistical association tests. The results supported our findings: using *APOE* burden as opposed to *APOE*-ε4 status provided better diagnostic prediction, however, adding PRSs to models already using *APOE* burden yielded only marginal gains (Supplementary Table 2). CSF tau and p-tau were better predicted by adding PHS or PRS1 to the models, but it was not the case for CSF Aβ (Supplementary Table 3). Lastly, there was no predictive gain by using polygenic scores on clinical progression in the survival analysis (Supplementary Table 6).

One limitation of this study was that, owing to sample overlap, the entire ADNI cohort could not be used for the PHS and PRS comparison. However, in order to demonstrate the effect of being *APOE*-ε4 homozygous, we tested *APOE*-ε4 burden in addition to *APOE*-ε4 status and compared the results to PHS on the entire cohort, finding broadly consistent results.

In summary, this work presents a comprehensive comparison of PHS, PRS and PRS-cs and their relation to a range of Alzheimer’s disease-related traits within the ADNI cohort. The results showed that there are not many effective differences between PHS and PRS1 in terms of associations with disease stage or biomarkers. In fact, many associations of PHS reported in earlier works were inflated due to lack of adjustment for the *APOE* locus, clinical diagnosis or underspecified statistical models. Given the ease with which PRS can be derived (i.e. from publicly available summary statistics) compared with PHS (i.e. requires reanalysis of the GWAS data), we believe the PRS constitutes the more practical choice. Moreover, on many of the tested traits, the major driving factor remained the *APOE* locus, with the sole exception of quantitative CSF-tau and p-tau measures.

## Funding

A.A. holds a Medical Research Council eMedLab Medical Bioinformatics Career Development Fellowship. This work was supported by the Medical Research Council [grant number MR/L016311/1]. M.A.S. acknowledges financial support the Engineering and Physical Sciences Research Council (EPSRC)-funded UCL Centre for Doctoral Training in Medical Imaging (EP/L016478/1). L.M.A. has received funding European Union’s Horizon 2020 research and innovation programme under grant agreement No. 666992. E.d.S. acknowledges supported from the National Institute for Health University College London Hospitals Biomedical Centre. D.M.C. is supported by grants from the Alzheimer Society (AS-PG-15-025), Alzheimer’s Research UK (ARUK-PG2014-1946) and Medical Research Council UK (MR/M023664/1). J.H. was supported in part by the Dolby Family Fund, the UK Dementia Research Institute and the Health University College London Hospitals Biomedical Centre as well as by an anonymous foundation. J.M.S. acknowledges the support of the National Institute for Health Research University College London Hospitals Biomedical Research Centre, Wolfson Foundation, Engineering and Physical Sciences Research Council (EPSRC) (EP/J020990/1), Medical Research Council (MRC) Dementias Platform UK (MR/L023784/1), Alzheimer’s Research UK (ARUK-PG2017-1946), Brain Research UK (UCC14191), Weston Brain Institute (UB170045) and European Union’s Horizon 2020 research and innovation programme (Grant 666992).

Data collection and sharing for this project were funded by the Alzheimer’s Disease Neuroimaging Initiative (ADNI; National Institutes of Health Grant U01 AG024904) and DOD ADNI (Department of Defense award number W81XWH-12-2-0012). ADNI is funded by the National Institute on Aging, the National Institute of Biomedical Imaging and Bioengineering, and through generous contributions from the following: AbbVie, Alzheimer’s Association; Alzheimer’s Drug Discovery Foundation; Araclon Biotech; BioClinica, Inc.; Biogen; Bristol-Myers Squibb Company; CereSpir, Inc.; Cogstate; Eisai Inc.; Elan Pharmaceuticals, Inc.; Eli Lilly and Company; EuroImmun; F. Hoffmann-La Roche Ltd and its affiliated company Genentech, Inc.; Fujirebio; GE Healthcare; IXICO Ltd.; Janssen Alzheimer Immunotherapy Research & Development, LLC.; Johnson & Johnson Pharmaceutical Research & Development LLC.; Lumosity; Lundbeck; Merck & Co., Inc.; Meso Scale Diagnostics, LLC.; NeuroRx Research; Neurotrack Technologies; Novartis Pharmaceuticals Corporation; Pfizer Inc.; Piramal Imaging; Servier; Takeda Pharmaceutical Company; and Transition Therapeutics. The Canadian Institutes of Health Research is providing funds to support ADNI clinical sites in Canada. Private sector contributions are facilitated by the Foundation for the National Institutes of Health (www.fnih.org). The grantee organization is the Northern California Institute for Research and Education, and the study is coordinated by the Alzheimer’s Therapeutic Research Institute at the University of Southern California. ADNI data are disseminated by the Laboratory for Neuro Imaging at the University of Southern California.

## Competing interests

J.M.S. has received research funding and PET tracer from AVID Radiopharmaceuticals (a wholly owned subsidiary of Eli Lilly); has consulted for Roche, Eli Lilly, Biogen and Merck, GE; received royalties from Oxford University Press and Henry Stewart Talks; given education lectures sponsored by Eli Lilly, Biogen and GE; and serves on a Data Safety Monitoring Committee for Axon Neuroscience SE.

## Supplementary Material

fcz047_Supplementary_Data

## Appendix: ADNI collaborators

**Part A: Leadership and Infrastructure**

**Principal Investigator**

Michael W. Weiner, MD UC San Francisco

**ATRI PI and Director of Coordinating Center Clinical Core**

Paul Aisen, MD University of Southern California

**Executive Committee**

Michael Weiner, MD UC San Francisco

Paul Aisen, MD University of Southern California

Ronald Petersen, MD, PhD Mayo Clinic, Rochester

Clifford R. Jack, Jr., MD Mayo Clinic, Rochester

William Jagust, MD UC Berkeley

John Q. Trojanowki, MD, PhD U Pennsylvania

Arthur W. Toga, PhD USC

Laurel Beckett, PhD UC

Davis Robert C. Green, MD, MPH Brigham and Women’s Hospital/Harvard Medical School

Andrew J. Saykin, PsyD Indiana University

John Morris, MD Washington University St. Louis

Leslie M. Shaw University of Pennsylvania

**ADNI External Advisory Board (ESAB)**

Zaven Khachaturian, PhD Prevent Alzheimer’s Disease 2020 (Chair)

Greg Sorensen, MD Siemens

Maria Carrillo, PhD Alzheimer’s Association

Lew Kuller, MD University of Pittsburgh

Marc Raichle, MD Washington University St. Louis

Steven Paul, MD Cornell University

Peter Davies, MD Albert Einstein College of Medicine of Yeshiva University

Howard Fillit, MD AD Drug Discovery Foundation

Franz Hefti, PhD Acumen Pharmaceuticals

David Holtzman, MD Washington University St. Louis

M. Marcel Mesulam, MD Northwestern University

William Potter, MD National Institute of Mental Health

Peter Snyder, PhD Brown University

**ADNI 3 Private Partner Scientific Board (PPSB)**

Veronika Logovinsky, MD, PhD Eli Lilly (Chair)

**Data and Publications Committee**

Robert C. Green, MD, MPH BWH/HMS (Chair)

**Resource Allocation Review Committee**

Tom Montine, MD, PhD University of Washington (Chair)

**Clinical Core Leaders**

Ronald Petersen, MD, PhD Mayo Clinic, Rochester (Core PI)

Paul Aisen, MD University of Southern California

**Clinical Informatics and Operations**

Gustavo Jimenez, MBS USC

Michael Donohue, PhD USC

Devon Gessert, BS USC

Kelly Harless, BA USC

Jennifer Salazar, MBS USC

Yuliana Cabrera, BS USC

Sarah Walter, MSc USC

Lindsey Hergesheimer, BS USC

**Biostatistics Core Leaders and Key Personnel**

Laurel Beckett, PhD UC Davis (Core PI)

Danielle Harvey, PhD UC Davis

Michael Donohue, PhD UC San Diego

**MRI Core Leaders and Key Personnel**

Clifford R. Jack, Jr., MD Mayo Clinic, Rochester (Core PI)

Matthew Bernstein, PhD Mayo Clinic, Rochester

Nick Fox, MD University of London

Paul Thompson, PhD UCLA School of Medicine

Norbert Schuff, PhD UCSF MRI

Charles DeCArli, MD UC Davis

Bret Borowski, RT Mayo Clinic

Jeff Gunter, PhD Mayo Clinic

Matt Senjem, MS Mayo Clinic

Prashanthi Vemuri, PhD Mayo Clinic

David Jones, MD Mayo Clinic

Kejal Kantarci Mayo Clinic

Chad Ward Mayo Clinic

**PET Core Leaders and Key Personnel**

William Jagust, MD UC Berkeley (Core PI)

Robert A. Koeppe, PhD University of Michigan

Norm Foster, MD University of Utah

Eric M. Reiman, MD Banner Alzheimer’s Institute

Kewei Chen, PhD Banner Alzheimer’s Institute

Chet Mathis, MD University of Pittsburgh

Susan Landau, PhD UC Berkeley

**Neuropathology Core Leaders**

John C. Morris, MD Washington University St. Louis

Nigel J. Cairns, PhD, FRCPath Washington University St. Louis

Erin Franklin, MS, CCRP Washington University St. Louis

Lisa Taylor-Reinwald, BA, HTL Washington University St. Louis (ASCP)—Past Investigator

**Biomarkers Core Leaders and Key Personnel**

Leslie M. Shaw, PhD UPenn School of Medicine

John Q. Trojanowki, MD, PhD UPenn School of Medicine

Virginia Lee, PhD, MBA UPenn School of Medicine

Magdalena Korecka, PhD UPenn School of Medicine

Michal Figurski, PhD UPenn School of Medicine

**Informatics Core Leaders and Key Personnel**

Arthur W. Toga, PhD USC (Core PI)

Karen Crawford USC

Scott Neu, PhD USC

**Genetics Core Leaders and Key Personnel**

Andrew J. Saykin, PsyD Indiana University

Tatiana M. Foroud, PhD Indiana University

Steven Potkin, MD UC UC Irvine

Li Shen, PhD Indiana University

Kelley Faber, MS, CCRC Indiana University

Sungeun Kim, PhD Indiana University

Kwangsik Nho, PhD Indiana University

**Initial Concept Planning & Development**

Michael W. Weiner, MD UC San Francisco

Lean Thal, MD UC San Diego

Zaven Khachaturian, PhD Prevent Alzheimer’s Disease 2020

**Early Project Proposal Development**

Leon Thal, MD UC San Diego

Neil Buckholtz National Institute on Aging

Michael W. Weiner, MD UC San Francisco

Peter J. Snyder, PhD Brown University

William Potter, MD National Institute of Mental Health

Steven Paul, MD Cornell University

Marilyn Albert, PhD Johns Hopkins University

Richard Frank, MD, PhD Richard Frank Consulting

Zaven Khachaturian, PhD Prevent Alzheimer’s Disease 2020

**NIA**

John Hsiao, MD National Institute on Aging

**Part B: Investigators by Site**

**Oregon Health & Science University:**

Joseph Quinn, MD

Lisa C. Silbert, MD

Betty Lind, BS

Jeffrey A. Kaye, MD, A.—Past Investigator

Raina Carter, BA—Past Investigator

Sara Dolen, BS—Past Investigator

**University of Southern California:**

Lon S. Schneider, MD

Sonia Pawluczyk, MD

Mauricio Becerra, BS

Liberty Teodoro, RN

Bryan M. Spann, DO, PhD—Past Investigator

**University of California—San Diego:**

James Brewer, MD, PhD

Helen Vanderswag, RN

Adam Fleisher, MD—Past Investigator

**University of Michigan:**

Jaimie Ziolkowski, MA, BS, TLLP

Judith L. Heidebrink, MD, MS

Joanne L. Lord, LPN, BA, CCRC—Past Investigator

**Mayo Clinic, Rochester:**

Ronald Petersen, MD, PhD

Sara S. Mason, RN

Colleen S. Albers, RN

David Knopman, MD

Kris Johnson, RN—Past Investigator

**Baylor College of Medicine:**

Javier Villanueva-Meyer, MD

Valory Pavlik, PhD

Nathaniel Pacini, MA

Ashley Lamb, MA

Joseph S. Kass, MD, LD, FAAN

Rachelle S. Doody, MD, PhD—Past Investigator

Victoria Shibley, MS—Past Investigator

Munir Chowdhury, MBBS, MS—Past Investigator

Susan Rountree, MD—Past Investigator

Mimi Dang, MD—Past Investigator

**Columbia University Medical Center:**

Yaakov Stern, PhD

Lawrence S. Honig, MD, PhD

Karen L. Bell, MD

Randy Yeh, MD

**Washington University, St. Louis:**

Beau Ances, MD, PhD, MSc

John C. Morris, MD

David Winkfield, BS

Maria Carroll, RN, MSN, GCNS-BC

Angela Oliver, RN, BSN, MSG

Mary L. Creech, RN, MSW—Past Investigator

Mark A. Mintun, MD—Past Investigator

Stacy Schneider, APRN, BC, GNP—Past Investigator

**University of Alabama—Birmingham:**

Daniel Marson, JD, PhD

David Geldmacher, MD

Marissa Natelson Love, MD

Randall Griffith, PhD, ABPP—Past Investigator

David Clark, MD—Past Investigator

John Brockington, MD—Past Investigator

**Mount Sinai School of Medicine:**

Hillel Grossman, MD

Effie Mitsis, PhD—Past Investigator

**Rush University Medical Center:**

Raj C. Shah, MD

Melissa Lamar, PhD

Patricia Samuels

**Wien Center:**

Ranjan Duara, MD

Maria T. Greig-Custo, MD

Rosemarie Rodriguez, PhD

**Johns Hopkins University:**

Marilyn Albert, PhD

Chiadi Onyike, MD

Daniel D’Agostino II, BS

StepMohammed O. Sheikh, MD

Jamika Singleton-Garvin, CCRP

Anaztasia Ulysse

Mrunalini Gaikwad

**Duke University Medical Center:**

P. Murali Doraiswamy, MBBS, FRCP

Jeffrey R. Petrella, MD

Olga James, MD

Salvador Borges-Neto, MD

Terence Z. Wong, MD—Past Investigator

Edward Coleman—Past Investigator

**University of Pennsylvania:**

Jason H. Karlawish, MD

David A. Wolk, MD

Sanjeev Vaishnavi, MD

Christopher M. Clark, MD—Past Investigator

Steven E. Arnold, MD—Past Investigator

**University of Kentucky:**

Charles D. Smith, MD

Greg Jicha, MD

Peter Hardy, PhD

Riham El Khouli, MD

Elizabeth Oates, MD

Gary Conrad, MD

**University of Pittsburgh:**

Oscar L. Lopez, MD

MaryAnn Oakley, MA

Donna M. Simpson, CRNP, MPH

**University of Rochester Medical Center:**

Anton P. Porsteinsson, MD

Kim Martin, RN

Nancy Kowalksi, MS, RNC

Melanie Keltz, RN

Bonnie S. Goldstein, MS, NP—Past Investigator

Kelly M. Makino, BS—Past Investigator

M. Saleem Ismail, MD—Past Investigator

Connie Brand, RN—Past Investigator

**University of California Irvine IMIND:**

Gaby Thai, MD

Aimee Pierce, MD

Beatriz Yanez, RN

Elizabeth Sosa, PhD

Megan Witbracht, PhD

**University of Texas Southwestern Medical School:**

Kyle Womack, MD

Dana Mathews, MD, PhD

Mary Quiceno, MD

**Emory University:**

Allan I. Levey, MD, PhD

James J. Lah, MD, PhD

Janet S. Cellar, DNP, PMHCNS-BC

**University of Kansas, Medical Center:**

Jeffrey M. Burns, MD

Russell H. Swerdlow, MD

William M. Brooks, PhD

**University of California, Los Angeles:**

Ellen Woo, PhD

Daniel H.S. Silverman, MD, PhD

Edmond Teng, MD, PhD

Sarah Kremen, MD

Liana Apostolova, MD—Past Investigator

Kathleen Tingus, PhD—Past Investigator

Po H. Lu, PsyD—Past Investigator

George Bartzokis, MD—Past Investigator

**Mayo Clinic, Jacksonville:**

Neill R Graff-Radford, MBBCH, FRCP (London)

Francine Parfitt, MSH, CCRC

Kim Poki-Walker, BA

**Indiana University:**

Martin R. Farlow, MD

Ann Marie Hake, MD

Brandy R. Matthews, MD—Past Investigator

Jared R. Brosch, MD

Scott Herring, RN, CCRC

**Yale University School of Medicine:**

Christopher H. van Dyck, MD

Richard E. Carson, PhD

Pradeep Varma, MD

**McGill Univ., Montreal-Jewish General Hospital:**

Howard Chertkow, MD

Howard Bergman, MD

Chris Hosein, MEdhanie Kielb, BS—Past Investigator

**Sunnybrook Health Sciences, Ontario:**

Sandra Black, MD, FRCPC

Bojana Stefanovic, PhD

Chris (Chinthaka) Heyn, BSC, PhD, MD, FRCPC

**U.B.C. Clinic for AD & Related Disorders:**

Ging-Yuek Robin Hsiung, MD, MHSc, FRCPC

Benita Mudge, BS

Vesna Sossi, PhD

Howard Feldman, MD, FRCPC—Past Investigator

Michele Assaly, MA—Past Investigator

**Cognitive Neurology—St. Joseph's, Ontario:**

Elizabeth Finger, MD

Stephen Pasternack, MD, PhD

William Pavlosky, MD

Irina Rachinsky, MD—Past Investigator

Dick Drost, PhD—Past Investigator

Andrew Kertesz, MD—Past Investigator

**Cleveland Clinic Lou Ruvo Center for Brain Health:**

Charles Bernick, MD, MPH

Donna Munic, PhD

**Northwestern University:**

Marek-Marsel Mesulam, MD

Emily Rogalski, PhD

Kristine Lipowski, MA

Sandra Weintraub, PhD

Borna Bonakdarpour, MD

Diana Kerwin, MD—Past Investigator

Chuang-Kuo Wu, MD, PhD—Past Investigator

Nancy Johnson, PhD—Past Investigator

**Premiere Research Inst (Palm Beach Neurology):**

Carl Sadowsky, MD

Teresa Villena, MD

**Georgetown University Medical Center:**

Raymond Scott Turner, MD, PhD

Kathleen Johnson, NP

Brigid Reynolds, NP

**Brigham and Women's Hospital:**

Reisa A. Sperling, MD

Keith A. Johnson, MD

Gad A. Marshall, MD

**Stanford University:**

Jerome Yesavage, MD

Joy L. Taylor, PhD

Steven Chao, MD, PhD

Barton Lane, MD—Past Investigator

Allyson Rosen, PhD—Past Investigator

Jared Tinklenberg, MD—Past Investigator

**Banner Sun Health Research Institute:**

Edward Zamrini, MD

Christine M. Belden, PsyD

Sherye A. Sirrel, CCRC

**Boston University:**

Neil Kowall, MD

Ronald Killiany, PhD

Andrew E. Budson, MD

Alexander Norbash, MD—Past Investigator

Patricia Lynn Johnson, BA—Past Investigator

**Howard University:**

Thomas O. Obisesan, MD, MPH

Ntekim E. Oyonumo, MD, PhD

Joanne Allard, PhD

Olu Ogunlana, BPharm

**Case Western Reserve University:**

Alan Lerner, MD

Paula Ogrocki, PhD

Curtis Tatsuoka, PhD

Parianne Fatica, BA, CCRC

**University of California, Davis—Sacramento:**

Evan Fletcher, PhD

Pauline Maillard, PhD

John Olichney, MD

Charles DeCarli, MD

Owen Carmichael, PhD—Past Investigator

**Neurological Care of CNY:**

Smita Kittur, MD—Past Investigator

**Parkwood Institute:**

Michael Borrie, MB ChB

T-Y Lee, PhD

Dr Rob Bartha, PhD

**University of Wisconsin:**

Sterling Johnson, PhD

Sanjay Asthana, MD

Cynthia M. Carlsson, MD, MS

**Banner Alzheimer's Institute:**

Pierre Tariot, MD

Anna Burke, MD

Joel Hetelle, BS

Kathryn DeMarco, BS

Nadira Trncic, MD, PhD, CCRC—Past Investigator

Adam Fleisher, MD—Past Investigator

Stephanie Reeder, BA—Past Investigator

**Dent Neurologic Institute:**

Vernice Bates, MD

Horacio Capote, MD

Michelle Rainka, PharmD, CCRP

**Ohio State University:**

Douglas W. Scharre, MD

Maria Kataki, MD, PhD

Rawan Tarawneh, MD

**Albany Medical College:**

Earl A. Zimmerman, MD

Dzintra Celmins, MD

David Hart, MD

**Hartford Hospital, Olin Neuropsychiatry Research Center:**

Godfrey D. Pearlson, MD

Karen Blank, MD

Karen Anderson, RN

**Dartmouth-Hitchcock Medical Center:**

Laura A. Flashman, PhD

Marc Seltzer, MD

Mary L. Hynes, RN, MPH

Robert B. Santulli, MD—Past Investigator

**Wake Forest University Health Sciences:**

Kaycee M. Sink, MD, MAS

Mia Yang, MD

Akiva Mintz, MD, PhD

**Rhode Island Hospital:**

Brian R. Ott, MD

Geoffrey Tremont, PhD

Lori A. Daiello, Pharm.D, ScM

**Butler Hospital:**

Stephen Salloway, MD, MS

Paul Malloy, PhD

Stephen Correia, PhD

Athena Lee, PhD

**UC San Francisco:**

Howard J. Rosen, MD

Bruce L. Miller, MD

David Perry, MD

**Medical University South Carolina:**

Jacobo Mintzer, MD, MBA

Kenneth Spicer, MD, PhD

David Bachman, MD

**St. Joseph’s Health Care:**

Elizabeth Finger, MD

Stephen Pasternak, MD

Irina Rachinsky, MD

John Rogers, MD

Andrew Kertesz, MD—Past Investigator

Dick Drost, MD—Past Investigator

**Nathan Kline Institute:**

Nunzio Pomara, MD

Raymundo Hernando, MD

Antero Sarrael, MD

**University of Iowa College of Medicine:**

Delwyn D. Miller, PharmD, MD

Karen Ekstam Smith, RN

Hristina Koleva, MD

Ki Won Nam, MD

Hyungsub Shim, MD

Susan K. Schultz, MD—Past Investigator

**Cornell University:**

Norman Relkin, MD, PhD

Gloria Chiang, MD

Michael Lin, MD

Lisa Ravdin, PhD

**University of South Florida: USF Health Byrd Alzheimer’s Institute:**

Amanda Smith, MD

Christi Leach, MD

Balebail Ashok Raj, MD—Past Investigator

Kristin Fargher, MD—Past Investigator

Courtney Bodge, PhD

## Abbreviations

Aβ: amyloid beta
ADGC: Alzheimer’s disease Genetics Consortium
ADNI: Alzheimer’s disease neuroimaging initiative
*APOE*: Apolipoprotein E gene
AUC: area under the ROC curve
BSI: boundary shift integral
CDR: clinical dementia rating
CN: cognitively normal
CSF: cerebrospinal fluid
EF: executive function
GWAS: genome-wide association study
LD: linkage disequilibrium
MAF: minor allele frequency
MCI: mild cognitive impairment
MEM: memory
PHS: polygenic hazard score
PRS: polygenic risk score
QC: quality control
ROC: Receiver Operating Characteristics
ROI: region of interest
SNP: single nucleotide polymorphism
SUVR: standardized uptake value ratio
