# Supplementary material for: A comprehensive analysis of methods for assessing polygenic burden on Alzheimer’s disease pathology and risk beyond APOE
Source: Brain Commun. 2019 Dec 16;2(1):fcz047. doi: 10.1093/braincomms/fcz047 (PMC7100005; doi:10.1093/braincomms/fcz047)
Supplement: fcz047_Supplementary_Data [file fcz047_supplementary_data.pdf]

Supplementary Material

A comprehensive analysis of methods for assessing polygenic burden on Alzheimer's disease pathology and risk beyond *APOE*

Andre Altmann[1], Marzia A Scelsi[1], Maryam Shoai[2,3], Eric de Silva[1,4], Leon M Aksman[1], David M Cash[3,5], John Hardy[2,3], Jonathan M Schott[3,5] for the Alzheimer's Disease Neuroimaging Initiative*

1 Centre for Medical Image Computing (CMIC), Department of Medical Physics and Biomedical Engineering, University College London (UCL), London, United Kingdom
2 Reta Lilla Research Laboratories, Department of Neurodegeneration, Queen Square Institute of Neurology, University College London (UCL), London, United Kingdom
3 UK Dementia Research Institute, University College London (UCL), London, United Kingdom
4 Institute for Health Informatics, University College London (UCL), London, United Kingdom
5 Dementia Research Centre, Queen Square Institute of Neurology, University College London (UCL), London, United Kingdom

*Data used in preparation of this article were obtained from the Alzheimer's Disease Neuroimaging Initiative (ADNI) database (adni.loni.usc.edu). As such, the investigators within the ADNI contributed to the design and implementation of ADNI and/or provided data but did not participate in analysis or writing of this report. A complete listing of ADNI investigators can be found at: http://adni.loni.usc.edu/wp-content/uploads/how_to_apply/ADNI_Acknowledgement_List.pdf

Corresponding author:
Dr. Andre Altmann
90 High Holborn
London WC1V 6LJ, 1st floor
Email: a.altmann@ucl.ac.uk

Supplementary Methods

We sought to further investigate the link between PRS1 and CSF tau levels by testing the overlap between genes contributing to the PRS and the tau's protein interaction network. First, we mapped the 55 SNPs that contribute to PRS1 to protein coding genes using FUMA (SNP2GENE) v1.3.5 based on SNP position and eQTL information with default settings (Watanabe *et al.*, 2017) (https://fuma.ctglab.nl/snp2gene/). Data for eQTL-based mapping was limited to brain tissues provided by GTEx v7. Next, we extracted a protein-protein interaction network for tau using the GeneMANIA webtool (Warde-Farley *et al.*, 2010) (https://genemania.org/). We used *MAPT*, i.e., the gene encoding the tau protein, as the single query gene and restricted the connections type to physical connections. Gene names of the top 100 interaction partners (i.e., the maximum number of interaction partners accessible through the web interface) were used as tau's protein interaction network. We tested for an over-representation of PRS1-contributing genes in the tau interaction network using Fisher's exact test. As the background gene list, we used 21,930 human refSeq genes (microRNAs, long non-coding RNAs, pseudo genes and antisense RNAs were excluded).

Supplementary Results

FUMA mapped the 55 SNPs of PRS1 to 86 genes (PRS1-contributing genes). A total of four genes (*APOE, BIN1, CKAP5, PTK2B)* were shared between the PRS1 contributing genes and the tau protein interaction network. This overlap was significantly higher than expected by chance (OR=12.5, P=4.1e-04).

Supplementary References

Warde-Farley, D. *et al.* The GeneMANIA prediction server: biological network integration for gene prioritization and predicting gene function. *Nucleic Acids Res* 2010, **38,** W214–W220.

Watanabe, K., Taskesen, E., van Bochoven, A. & Posthuma, D. Functional mapping and annotation of genetic associations with FUMA. *Nat Commun* 2017, **8,** 1826.

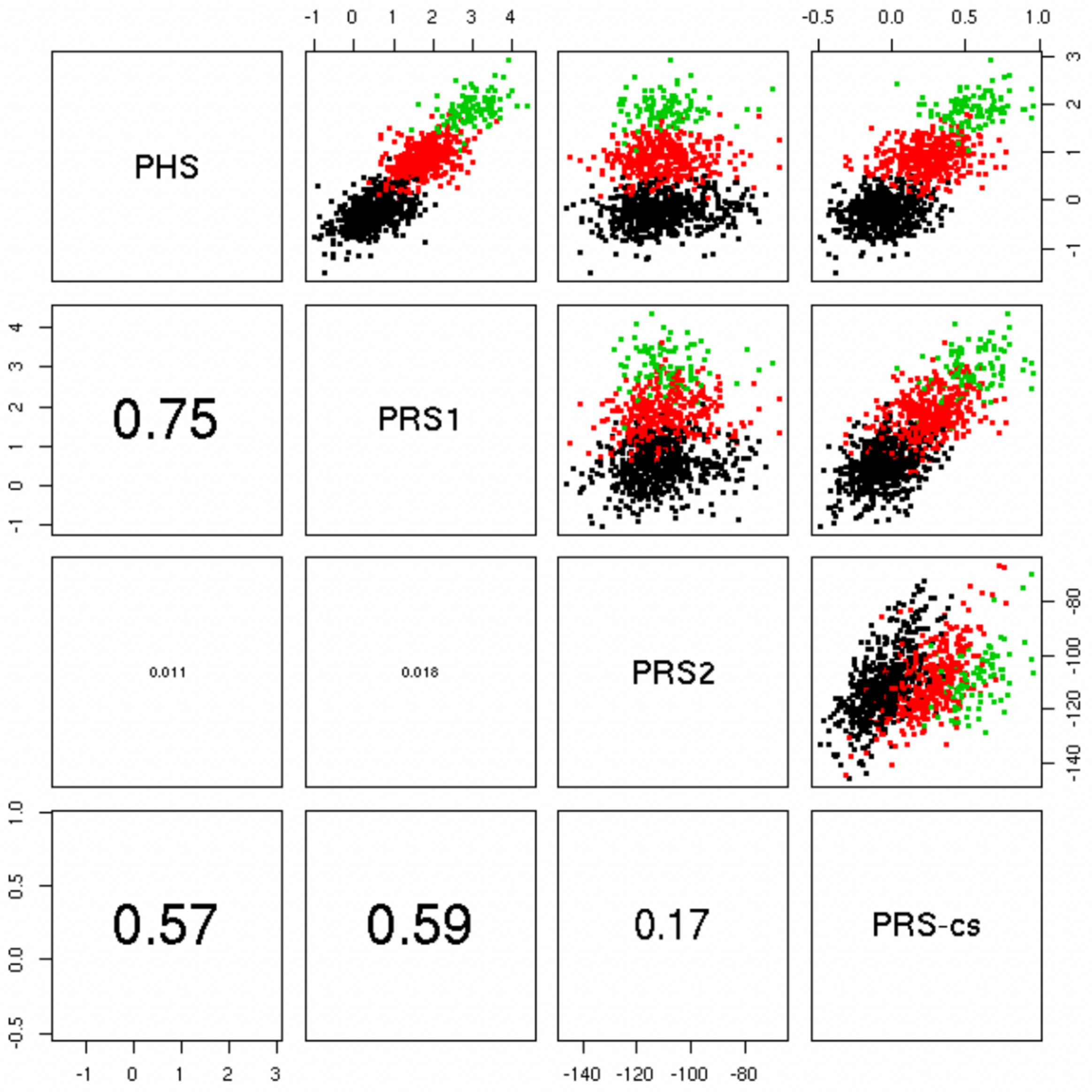

<u>Supplementary Figure 1:</u> Correlation between different polygenic scores on ADNI subjects. Pairwise scatter plots and correlations between the four polygenic scores for the n=994 ADNI participants remaining in the analysis after QC. Labels on the diagonal indicate the type of polygenic score, the panels in the lower triangle display the correlation (r^2) between two given scores and the panels in the upper triangle depict the scatter plot. Colors indicate the number of ε4 alleles (0=black, 1=red, 2=green).

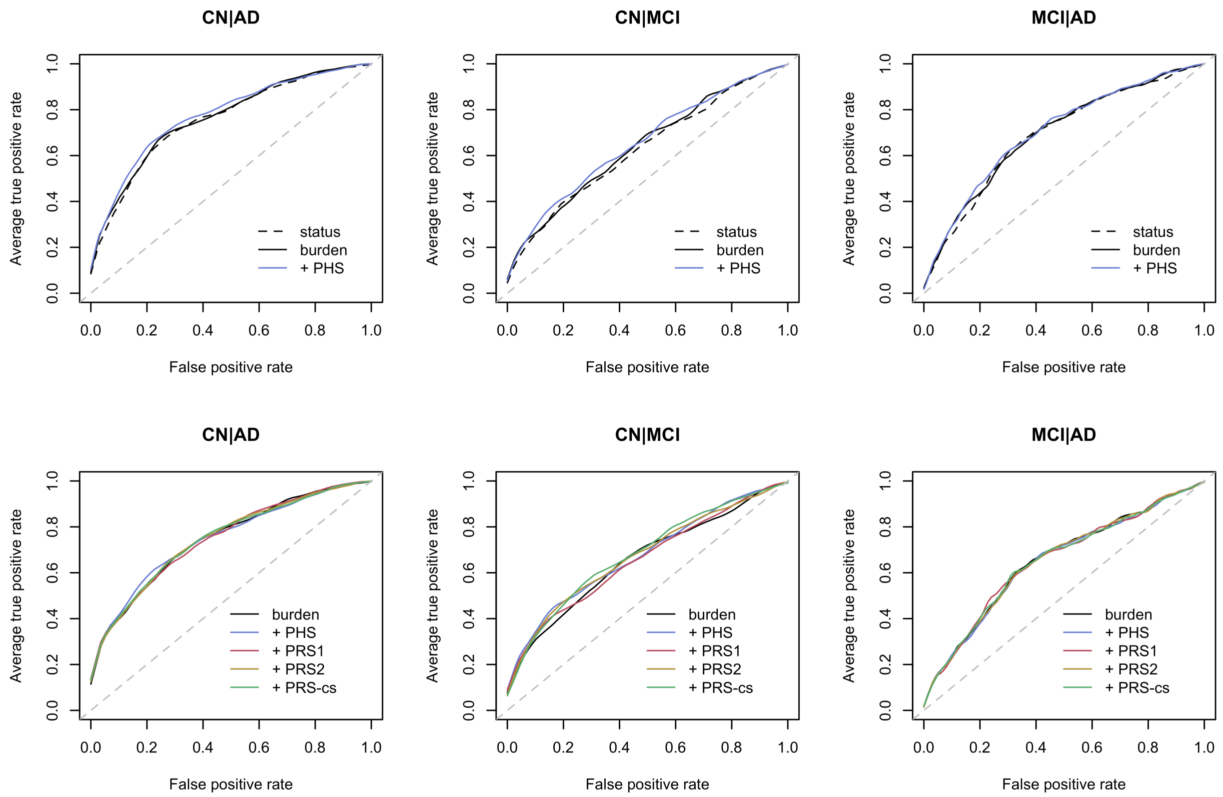

<u>Supplementary Figure 2</u>: Averaged ROC curves from 10-fold cross-validation. Logistic regression models were trained to classify different diagnoses pairs (corresponding to columns) using as input age (at diagnosis), sex, education and either *APOE* ε4 "status" or *APOE* "burden". To these baseline models the different polygenic scores were added. Performance was assessed in a 10-fold cross-validation setting and the ROC curves were averaged over these ten folds; the grey dashed line represents random performance. Panels in the top row utilize the entire cohort while panels in the bottom row were generated on the reduced cohort. Different polygenic scores are represented by different colors.

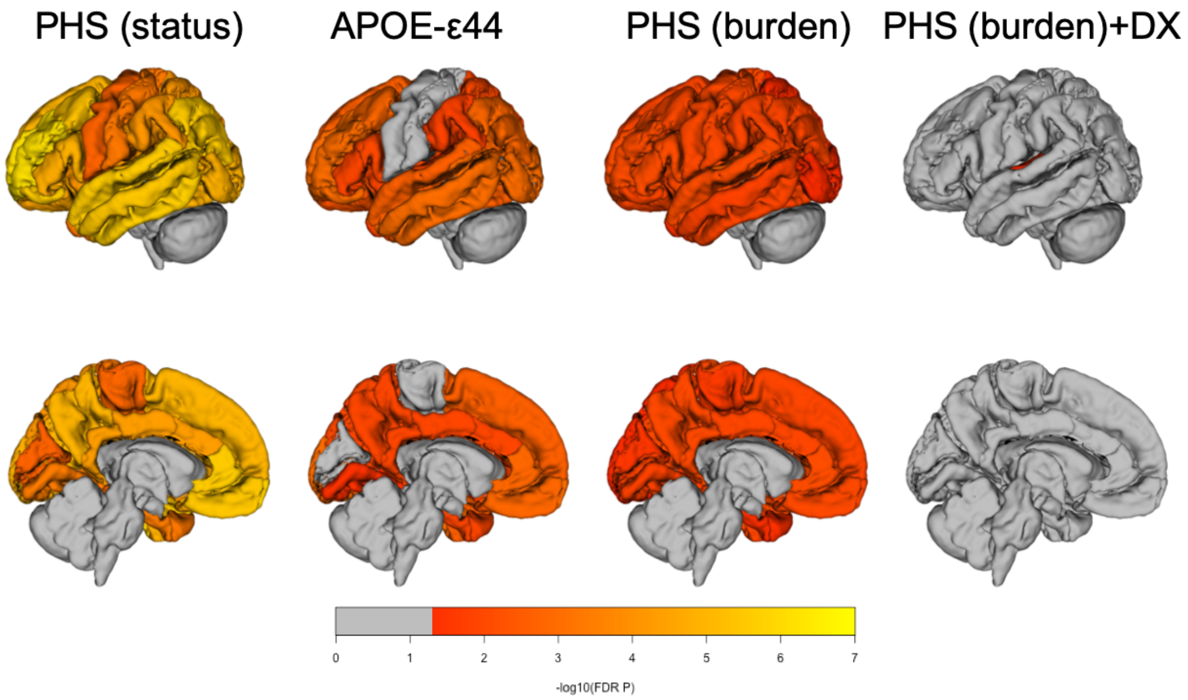

<u>Supplementary Figure 3:</u> Regional associations between polygenic risk scores and regional uptake of amyloid. The color code represents the -log10 FDR-corrected p-values obtained from linear models adjusted for age, sex, education and *APOE* (either for "status" or for "burden"). The model in the rightmost column was stratified by disease group at the time of imaging.

Supplementary Table 1: Baseline demographics of the reduced cohort. Comprising n=994 individuals after quality control of the genetic data and who did not contribute to the ADGC study. Age given as mean, MMSE given as median, Years of education as mean, amyloid PET as negative (-) or positive (+) and unavailable (NA).

	CN	MCI	AD	total	P-value (F-value)
N	264	581	149	994	
Female (%)	141 (53)	233 (40)	63 (42)	438 (44)	0.001 (6.692)
age	73.7 (5.8)	72.7 (7.7)	74.1 (8.4)	73.2 (7.3)	0.0557 (2.896)
APOE-e4 (0/1/2)	184/74/6	302/219/60	46/71/32	532/364/98	<2e-16 (39.65)
MMSE (IQR)	29 (29-30)	28 (27-29)	23 (21-25)	28 (26-29)	<2e-16 (643.5)
Education	16.62 (2.63)	16.15 (2.82)	15.88 (2.68)	16.24 (2.76)	0.016 (4.147)
Amyloid PET (-/+/NA)	146/76/42	171/216/194	14/107/28	331/399/265	<2e-16 (53.24)

<u>Supplementary Table 2</u>: Predictive performance for classifying clinical diagnosis. Logistic regression models were used to predict patients' diagnosis. All models used age (at diagnosis), sex and education as features. The "status" model used *APOE* ε4 carrier status while the "burden" model used allele counts for *APOE* ε2 and ε4. Polygenic scores were added to either the status or the burden model (indicated by +). Predictive out-of-sample performance was estimated using 10-fold cross-validation. Performance was measured as area under the ROC curve (AUC). For each setting the mean and standard deviation (sd) of the AUC from the 10 folds are provided. The burden model was compared to the status model, and models using a polygenic score were compared to the corresponding burden or status model. P-values were computed using a signed paired Wilcoxon rank sum test on the ten pairs of AUC measures.

		**CN | AD**			**CN | MCI**			**MCI | AD**			**CN | MCI + AD**		
		**mean**	**sd**	**p-value**	**mean**	**sd**	**p-value**	**mean**	**sd**	**p-value**	**mean**	**sd**	**p-value**
Entire cohort	status	0.758	0.044	NA	0.629	0.076	NA	0.69	0.045	NA	0.695	0.04	NA
	+PHS	0.777	0.044	0.0049	0.658	0.078	0.0064	0.705	0.042	0.00195	0.718	0.036	0.0049
	burden	0.765	0.044	0.065	0.642	0.083	0.0068	0.694	0.035	0.19	0.704	0.039	0.042
	+PHS	0.777	0.044	0.0029	0.654	0.081	0.053	0.702	0.039	0.065	0.715	0.036	0.042
Reduced cohort	status	0.735	0.069	NA	0.649	0.075	NA	0.642	0.052	NA	0.677	0.044	NA
	burden	0.749	0.059	0.032	0.659	0.083	0.065	0.654	0.06	0.00977	0.689	0.04	0.042
	+PHS	0.751	0.066	0.32	0.677	0.083	0.014	0.649	0.057	0.93	0.7	0.035	0.019
	+PRS1	0.75	0.057	0.55	0.659	0.09	0.46	0.653	0.056	0.42	0.695	0.044	0.08
	+PRS2	0.751	0.059	0.38	0.674	0.066	0.065	0.653	0.06	0.85	0.702	0.043	0.053
	+PRS-cs	0.751	0.063	0.31	0.682	0.07	0.053	0.652	0.062	0.59	0.709	0.042	0.0029

<u>Supplementary Table 3</u>: Predictive performance for CSF biomarker levels. Linear regression models were used to predict patients' CSF biomarker levels. All models used diagnosis (at study entry), age, sex and education as features. The "status" model used *APOE* ε4 carrier status while the "burden" model used allele counts for *APOE* ε2 and ε4. Polygenic scores were added to either the status or the burden model (indicated by +). Predictive out-of-sample performance was estimated using 10-fold cross-validation. Performance was measured as Pearson's correlation coefficient (*r*). For each setting the mean and standard deviation (sd) of the correlation coefficients from the 10 folds are provided. The burden model was compared to the status model, and models using a polygenic score were compared to the corresponding burden or status model. P-values were computed using a signed paired Wilcoxon rank sum test on the ten pairs of AUC measures.

		**tau**			**p-tau**			**Aβ**		
		**mean**	**sd**	**p-value**	**mean**	**sd**	**p-value**	**mean**	**sd**	**p-value**
Entire cohort	status	0.408	0.076	NA	0.435	0.07	NA	0.559	0.077	NA
	+PHS	0.422	0.093	0.08	0.448	0.087	0.08	0.574	0.078	0.0049
	burden	0.41	0.079	0.42	0.436	0.071	0.42	0.587	0.081	0.00098
	+PHS	0.417	0.093	0.19	0.442	0.087	0.24	0.586	0.08	0.95
Reduced cohort	status	0.422	0.097	NA	0.447	0.09	NA	0.548	0.073	NA
	burden	0.429	0.091	0.19	0.454	0.087	0.14	0.575	0.07	0.024
	+PHS	0.442	0.081	0.019	0.466	0.075	0.042	0.574	0.071	0.84
	+PRS1	0.447	0.076	0.042	0.472	0.07	0.065	0.573	0.068	0.62
	+PRS2	0.43	0.092	0.25	0.455	0.089	0.31	0.574	0.07	0.62
	+PRS-cs	0.428	0.092	0.65	0.454	0.088	0.46	0.573	0.071	0.35

Supplementary Table 4: Association of polygenic scores with regional amyloid burden on the full dataset. P-values are FDR-corrected. Significant p-values ($P_{FDR} < 0.05$) are highlighted in bold. * indicates additional stratification by diagnosis at the time of PET imaging.

ROI	PHS (status)	PHS (burden)	APOE-e44	PHS (burden)*
COMPOSITE	**2.89E-06**	**6.36E-03**	**1.69E-03**	8.27E-02
BANKSSTS	**1.69E-06**	**6.36E-03**	**6.65E-04**	8.27E-02
CAUDALANTERIORCINGULATE	**3.51E-05**	**8.98E-03**	**8.77E-03**	8.28E-02
CAUDALMIDDLEFRONTAL	**3.32E-06**	**6.36E-03**	**1.24E-03**	8.27E-02
CUNEUS	**1.32E-03**	**2.17E-02**	1.36E-01	1.06E-01
ENTORHINAL	**2.91E-04**	**3.80E-02**	**8.37E-03**	1.06E-01
FRONTALPOLE	**1.48E-06**	**9.44E-03**	**6.65E-04**	9.81E-02
FUSIFORM	**1.08E-05**	**9.44E-03**	**1.71E-03**	9.77E-02
INFERIORPARIETAL	**2.52E-06**	**9.14E-03**	**1.04E-03**	8.28E-02
INFERIORTEMPORAL	**1.69E-06**	**9.44E-03**	**6.65E-04**	1.01E-01
INSULA	**5.46E-05**	**6.36E-03**	**9.72E-03**	8.27E-02
ISTHMUSCINGULATE	**3.51E-05**	**6.91E-03**	**9.63E-03**	8.27E-02
LATERALOCCIPITAL	**9.76E-06**	**4.78E-02**	**7.51E-04**	1.55E-01
LATERALORBITOFRONTAL	**2.25E-05**	**9.44E-03**	**3.16E-03**	9.77E-02
LINGUAL	**2.91E-04**	**1.12E-02**	**3.05E-02**	9.77E-02
MEDIALORBITOFRONTAL	**2.89E-06**	**6.36E-03**	**1.24E-03**	8.28E-02
MIDDLETEMPORAL	**2.89E-06**	**1.17E-02**	**7.06E-04**	1.06E-01
PARACENTRAL	**1.12E-03**	**1.93E-02**	1.25E-01	9.77E-02
PARAHIPPOCAMPAL	**1.08E-03**	**1.01E-02**	9.40E-02	8.28E-02
PARSOPERCULARIS	**9.76E-06**	**5.84E-03**	**7.21E-03**	8.21E-02
PARSORBITALIS	**2.52E-06**	**8.98E-03**	**7.06E-04**	9.08E-02
PARSTRIANGULARIS	**1.40E-05**	**5.84E-03**	**8.31E-03**	8.21E-02

PERICALCARINE	**1.41E-03**	**1.02E-02**	1.67E-01	9.77E-02
POSTCENTRAL	**3.89E-04**	**6.36E-03**	1.36E-01	8.21E-02
POSTERIORCINGULATE	**3.12E-05**	**8.98E-03**	**9.72E-03**	9.08E-02
PRECENTRAL	**1.41E-03**	**6.68E-03**	1.96E-01	8.21E-02
PRECUNEUS	**8.83E-06**	**6.36E-03**	**8.31E-03**	8.27E-02
ROSTRALANTERIORCINGULATE	**2.52E-06**	**5.84E-03**	**1.24E-03**	8.27E-02
ROSTRALMIDDLEFRONTAL	**6.22E-07**	**5.84E-03**	**6.65E-04**	8.27E-02
SUPERIORFRONTAL	**8.83E-06**	**9.44E-03**	**1.71E-03**	9.77E-02
SUPERIORPARIETAL	**6.09E-05**	**4.42E-02**	**6.13E-03**	1.92E-01
SUPERIORTEMPORAL	**2.52E-06**	**5.84E-03**	**1.71E-03**	8.21E-02
SUPRAMARGINAL	**5.19E-05**	**9.44E-03**	**1.17E-02**	9.08E-02
TEMPORALPOLE	**3.52E-04**	**3.48E-02**	**8.37E-03**	1.10E-01
TRANSVERSETEMPORAL	**6.67E-06**	**1.40E-03**	**9.72E-03**	**4.44E-02**

Supplementary Table 5: Association of polygenic scores with regional amyloid burden on the reduced dataset. ADGC subjects were removed. All models were adjusted for *APOE* locus (ε2 and ε4 burden) and stratified by disease status. P-values are FDR-corrected. Significant p-values ($P_{FDR} < 0.05$) are highlighted in bold. * indicates additional correction for genetic population structure.

ROI	PHS (burden)	PRS1	PRS2	PRS2*	PRS-cs
COMPOSITE	5.36E-02	**3.81E-03**	8.83E-01	2.27E-01	8.80E-01
BANKSSTS	5.36E-02	**1.31E-02**	8.83E-01	2.27E-01	8.85E-01
CAUDALANTERIORCINGULATE	5.36E-02	**1.29E-02**	8.83E-01	1.97E-01	8.80E-01
CAUDALMIDDLEFRONTAL	5.36E-02	**3.81E-03**	8.83E-01	2.74E-01	8.85E-01
CUNEUS	1.55E-01	5.27E-01	9.46E-01	3.36E-01	9.37E-01
ENTORHINAL	6.44E-02	**6.32E-03**	8.83E-01	1.97E-01	9.37E-01
FRONTALPOLE	8.93E-02	**3.81E-03**	8.83E-01	1.97E-01	8.80E-01
FUSIFORM	1.00E-01	7.70E-02	8.83E-01	2.27E-01	9.37E-01
INFERIORPARIETAL	5.96E-02	**3.81E-03**	9.95E-01	2.74E-01	8.80E-01
INFERIORTEMPORAL	1.00E-01	**1.31E-02**	8.83E-01	2.27E-01	8.85E-01
INSULA	5.67E-02	**1.88E-02**	8.83E-01	1.97E-01	9.37E-01
ISTHMUSCINGULATE	5.36E-02	**3.81E-03**	8.83E-01	1.97E-01	8.80E-01
LATERALOCCIPITAL	2.15E-01	3.99E-01	8.83E-01	4.47E-01	8.85E-01
LATERALORBITOFRONTAL	7.14E-02	**3.81E-03**	8.83E-01	1.97E-01	8.80E-01
LINGUAL	1.43E-01	7.77E-01	9.72E-01	3.31E-01	8.85E-01
MEDIALORBITOFRONTAL	5.96E-02	**3.81E-03**	8.83E-01	1.97E-01	8.80E-01
MIDDLETEMPORAL	1.17E-01	**3.81E-03**	9.46E-01	2.74E-01	8.85E-01
PARACENTRAL	6.52E-02	5.05E-02	8.83E-01	2.27E-01	8.80E-01
PARAHIPPOCAMPAL	5.42E-02	**1.31E-02**	8.83E-01	2.28E-01	9.37E-01
PARSOPERCULARIS	5.36E-02	**3.81E-03**	8.83E-01	2.27E-01	8.80E-01

PARSORBITALIS	5.36E-02	**4.00E-04**	8.83E-01	2.27E-01	8.80E-01
PARSTRIANGULARIS	5.36E-02	**3.52E-03**	8.83E-01	2.28E-01	8.80E-01
PERICALCARINE	1.40E-01	6.68E-01	9.64E-01	2.74E-01	9.37E-01
POSTCENTRAL	5.36E-02	5.05E-02	9.95E-01	3.31E-01	8.80E-01
POSTERIORCINGULATE	5.79E-02	**3.81E-03**	8.83E-01	1.97E-01	8.80E-01
PRECENTRAL	5.36E-02	**4.46E-02**	9.95E-01	2.94E-01	8.85E-01
PRECUNEUS	5.36E-02	**3.81E-03**	8.83E-01	1.97E-01	8.80E-01
ROSTRALANTERIORCINGULATE	5.36E-02	**4.04E-03**	8.83E-01	1.97E-01	8.80E-01
ROSTRALMIDDLEFRONTAL	5.36E-02	**3.74E-03**	8.83E-01	2.27E-01	8.80E-01
SUPERIORFRONTAL	8.46E-02	**3.81E-03**	8.83E-01	2.28E-01	8.80E-01
SUPERIORPARIETAL	1.82E-01	6.06E-02	9.46E-01	2.94E-01	8.85E-01
SUPERIORTEMPORAL	5.36E-02	**4.01E-03**	8.83E-01	2.74E-01	8.85E-01
SUPRAMARGINAL	5.79E-02	**1.40E-02**	9.72E-01	3.31E-01	8.85E-01
TEMPORALPOLE	1.40E-01	**3.81E-03**	8.83E-01	2.94E-01	9.37E-01
TRANSVERSETEMPORAL	**3.80E-02**	**1.96E-02**	8.83E-01	2.27E-01	8.90E-01

<u>Supplementary Table 6</u>: Predictive performance for clinical conversion prediction. Cox regression models were used to predict patients' clinical conversion. All models used diagnosis (at study entry), age, sex and education as features. The "status" model used *APOE* ε4 carrier status while the "burden" model used allele counts for *APOE* ε2 and ε4. Polygenic scores were added to either the status or the burden model (indicated by +). Predictive out-of-sample performance was estimated using 10-fold cross-validation. Performance was measured as concordance index (c-index). For each setting the mean and standard deviation (sd) of the c-index from the 10 folds are provided. The burden model was compared to the status model, and models using a polygenic score were compared to the corresponding burden or status model. P-values were computed using a signed paired Wilcoxon rank sum test on the ten pairs of AUC measures.

		**mean**	**sd**	**p-value**
Entire cohort	status	0.777	0.031	NA
	+PHS	0.78	0.031	0.12
	burden	0.773	0.035	NA
	+PHS	0.777	0.031	0.08
Reduced cohort	status	0.745	0.069	NA
	burden	0.741	0.064	0.75
	+PHS	0.737	0.066	0.99
	+PRS1	0.737	0.063	0.98
	+PRS2	0.739	0.063	0.99
	PRS-cs	0.742	0.062	0.42

Supplementary Table 7: Model comparison between random intercepts only and random intercepts and random slopes linear mixed effects models for the PHS (status) score. Model Log Likelihood values for linear mixed effects models for change in clinical dementia rating sum of boxes (CDR-SB) and composite scores for memory and executive function, the X^2 for the model comparison and the corresponding P-value (2 degrees of freedom).

	CDR-SB	Memory	Executive function
Log Likelihood Random intercept	-4605.2	-1235.45	-2101.0
Log Likelihood Random intercept + slope	-3603.7	-977.62	-1816.2
X^2	2002.9	515.67	569.58
P-value	<2.2e-16	<2.2e-16	<2.2e-16
